# Secreted novel AID/APOBEC-like deaminase 1 (SNAD1) – a new important player in fish immunology

**DOI:** 10.3389/fimmu.2024.1340273

**Published:** 2024-03-27

**Authors:** Anna M. Majewska, Mariola A. Dietrich, Lucyna Budzko, Mikołaj Adamek, Marek Figlerowicz, Andrzej Ciereszko

**Affiliations:** ^1^ Department of Gamete and Embryo Biology, Institute of Animal Reproduction and Food Research, Polish Academy of Sciences, Olsztyn, Poland; ^2^ Department of Molecular and Systems Biology, Institute of Bioorganic Chemistry, Polish Academy of Sciences, Poznań, Poland; ^3^ Fish Disease Research Unit, Institute for Parasitology, University of Veterinary Medicine, Hannover, Germany

**Keywords:** secreted cytidine deaminase 1 (SNAD1), nucleic acids editing, fish, infection, immunology, immune responses

## Abstract

The AID/APOBECs are a group of zinc-dependent cytidine deaminases that catalyse the deamination of bases in nucleic acids, resulting in a cytidine to uridine transition. Secreted novel AID/APOBEC-like deaminases (SNADs), characterized by the presence of a signal peptide are unique among all of intracellular classical AID/APOBECs, which are the central part of antibody diversity and antiviral defense. To date, there is no available knowledge on SNADs including protein characterization, biochemical characteristics and catalytic activity. We used various *in silico* approaches to define the phylogeny of SNADs, their common structural features, and their potential structural variations in fish species. Our analysis provides strong evidence of the universal presence of SNAD1 proteins/transcripts in fish, in which expression commences after hatching and is highest in anatomical organs linked to the immune system. Moreover, we searched published fish data and identified previously, “uncharacterized proteins” and transcripts as SNAD1 sequences. Our review into immunological research suggests SNAD1 role in immune response to infection or immunization, and interactions with the intestinal microbiota. We also noted SNAD1 association with temperature acclimation, environmental pollution and sex-based expression differences, with females showing higher level. To validate *in silico* predictions we performed expression studies of several SNAD1 gene variants in carp, which revealed distinct patterns of responses under different conditions. Dual sensitivity to environmental and pathogenic stress highlights its importance in the fish and potentially enhancing thermotolerance and immune defense. Revealing the biological roles of SNADs represents an exciting new area of research related to the role of DNA and/or RNA editing in fish biology.

## 1 Introduction

**Figure 1:**
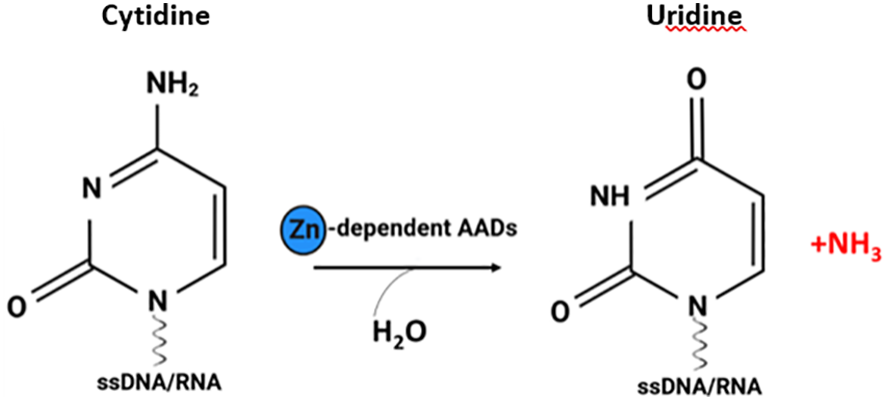
The cytosine deamination reaction catalysed by AADs. Zinc dependent AADs act on single-stranded DNA (ssDNA) and/or RNA, resulting in a cytidine-to-uridine transition (figure created with http://biorender.com).

Activation-induced cytidine deaminase/apolipoprotein B mRNA editing catalytic polypeptide-like deaminases (AID/APOBECs; AADs) are zinc-dependent deaminases that catalyse the deamination of bases in nucleic acids, resulting in a cytidine to uridine transition and change the genetic information converted by nucleic acids (**Figure 1**) (1). AAD family members, due to their ability to edit RNA and/or DNA sequences, catalyse a wide array of genomic and epigenomic modifications affecting various functions, including DNA and/or RNA mutator activity and the modulation of both innate and adaptive immune responses, with an important role in antibody diversification (2). Moreover, these enzymes are involved in restricting endogenous and exogenous retroviruses and participate in epigenetic regulation and lipid metabolism (3). To date all family of AID/APOBECs enzymes have been recognized as intracellular enzymes (3, 4). The most characterized members of classical AADs are AID and APOBECs [1, 2, 3 (A-D, F–H), 4] (5). For more details on the classification and functions of the classic AAD family members, we refer the readers to a few excellent recent reviews (4–6).

**Figure 2:**
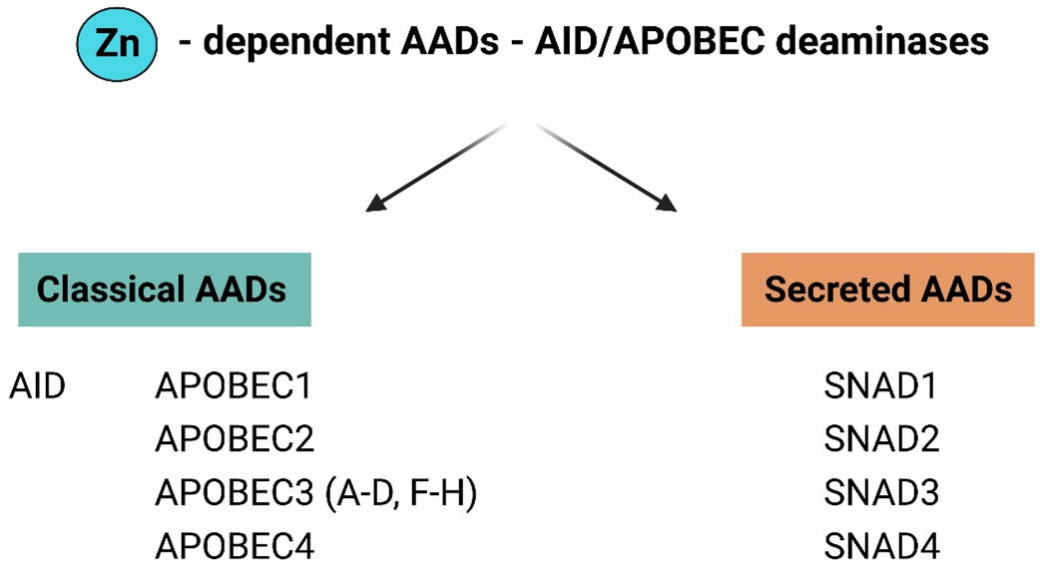
General division of AAD family members in to classical and secreted proteins. The AADs family have been divided into two major groups: classical AADs and Secreted deaminases (SNADs). The most characterized members of classical AADs are AID and APOBECs [1, 2, 3 (A-D, F–H), 4]. SNADs are the only AADs – like enzymes with a characteristic predicted secretion sequence and are divided into four major subclades SNADs 1–4. (Figure created with http://biorender.com).

In 2018, Krishnan et al. (4) performed DNA phylogenetic sequence analyses that revealed the presence of secreted deaminases, named secreted novel AID/APOBEC-like deaminases (SNADs), that are characterized by the presence of a signal peptide which distinguishes SNADs from intracellular “classic AADs”. The initial division of AADs into secreted deaminases (SNADs) and classic AADs occurred during early metazoan evolution, after which these enzymes diversified into various eukaryotic clades as a consequence of extensive structural alterations, sequence evolution, gene loss and lineage-specific expansion (LSE) (**Figure 2**) (4). SNADs appeared early in animal evolution; however, among vertebrates, they are present only in lower poikilothermic vertebrates (including fish) and have been lost in lineages that maintain a constant high body temperature, namely, birds, marsupials, and mammals (4). SNAD enzymes constitute a separate branch of the classical AAD family. SNAD1 appears throughout the poikilothermic vertebrate phylum, whereas SNAD2 and 3 appear only in ray-finned fishes and seem to have arisen via whole-genome duplication events and/or subsequent expansion of this branch. SNAD4 has only been identified in sponges (4, 5).

It should be emphasized that the secretory nature of SNAD enzymes makes them unique among AID-like enzymes in eukaryotes. Due to the importance of AADs in vertebrates as a central part of antibody diversity and antiviral defence, it is critical to understand the origin and role of secreted cytidine deaminases. To date, there is no available knowledge on SNAD including protein characterization, the biochemical characteristics and catalytic activity. The biological role of SNAD is completely unknown, and its unrevealing may identify a novel mechanism for cytidine deaminase action outside the cell.

In our previous study we provided, for the first time, experimental direct evidence confirming the presence of SNAD1 in carp (which was named at the time as cold acclimation protein 31, Cap31; hypothetical protein CypCar 00026018) (7). We performed cloning and sequencing of Cap31, exhibiting a notable response to cold acclimation (7), and after a multiple sequence alignment of the cloned Cap31, we conclusively identified this protein as SNAD1.

In this study, we aimed to expand our knowledge about SNADs in fish. We used various *in silico* approaches to define the phylogeny of SNADs, presented common structural features of SNAD1 members, and investigated their potential structural variations in fish species. Successful identification of homology between previously, “uncharacterized transcripts” and SNAD1 allows us to provide evidence of the universal presence of SNAD1 transcripts in fish and demonstrate their expression in different developmental stages and in anatomical organs linked to the immune system. Furthermore, using different protein and genomic databases we searched published fish data and identified previously, “uncharacterized proteins” and transcripts as SNAD1 sequences. Based on the literature we found evidence suggesting that these enzymes are involved in immunological responses and that SNAD1 may be related to temperature acclimation and the response to environmental pollution. Additionally, we showed that SNAD1 levels are influenced by sex, showing higher abundance in females. To confirm *in silico* predictions of SNAD1 relationship with temperature acclimation and both bacterial and viral infection, we performed expression studies of several SNAD1 gene variants, uncovering distinct patterns of responses under different conditions. This dual sensitivity highlights the importance of SNAD1 in the carp adaptation to environmental changes and pathogenic challenges, possibly contributing to thermotolerance and immune defense mechanisms. Revealing the biological roles of SNADs represents an exciting new area of research related to the role of RNA-editing enzymes in fish immunology.

## 2 Materials and methods

### 2.1 Phylogenetic analysis

Phylogenetic analysis of all available SNADs sequences in common carp (*Cyprinus carpio*) (GenBank ID: ADV68699; XP_042609327; XP_042609326; XP_018918723; XP_042610696; XP_042590618; XP_042627763; XP_018965652; XP_018976522; XP_042606770; XP042627685; XP_042596168; XP_042600158; XP_042596170; XP_042599546), zebrafish (*Danio rerio*) (GenBank ID: NP_001373174; NP_001373175; XP_021322222; NP_001373168) rainbow trout (*Oncorhynchus mykiss*) (GenBank ID: XP_036825965; XP_021439285; XP_036805675) and Atlantic salmon (*Salmo salar)* (XP_0140067042*)* were analyzed for their phylogenetic relationships to other SNAD sequences found in GenBank using the tools available at www.phylogeny.fr (8). Sequence alignment was conducted with the full mode of MUSCLE algorithm (9). Phylogenetic analyses, based on the maximum likelihood method, were performed by SH-like Approximate Likelihood-Ratio Test (aLRT) using the PhyML software (10). Subsequently, the phylogenetic tree was visualized and rendered using TreeDyn (11).

### 2.2 *In silico* gene structure and expression pattern

The genomic structure and function of SNAD1 genes was investigated by BLAST analysis of all SNAD1 LOCs. The following databases were checked: common carp reference genome ASM1834038v1 (GCF_018340385), zebrafish reference genome GRCz11 (GCF_000002035), rainbow trout reference genome USDA_OmykA_1.1 (GCF_013265735) and Atlantic salmon reference genome ICSASG_v2 (GCA_000233375). We also searched the expressed sequence tags (EST) and transcriptome shotgun assembly (TSA) sequence databases to check if the gene mRNA had been previously detected. In addition, we screened our in-house common carp blood and seminal plasma samples for the presence of SNAD1 proteins.

### 2.3 Predictions of SNAD1 secondary and tertiary structure

Multiple sequence alignment was conducted using the Clustal Omega program available at www.ebi.ac.uk/services (12) and visualize (including 2D structure) using ESPript 3.0 program (13) available at https://espript.ibcp.fr/ESPript/cgi-bin/ESPript.cgi. 3D structure models were predicted *ab initio* for selected SNAD1 members using the RoseTTAFold method (14) on the Robetta server (https://robetta.bakerlab.org/submit.php). Modelled structures were visualized and analysed using UCSF Chimera software (15). To identify signal peptides we used ProtterServer (16) available at www.wlab.ethz.ch/protter/start/. To generate superposition of 3D structures, we used Pairwise Structure Alignment (17, 18) tool available at www.rcsb.org/docs/tools/pairwise-structure-alignment.

### 2.4 SNAD1 gene expression at different developmental stages in zebrafish

The European Bioinformatics Institute Gene Expression Atlas (http://www.ebi.ac.uk/gxa/) was queried for baseline gene expression data for the si:dkey-96g2.1 gene (encoding a putative SNAD1 homolog) in different developmental stages of zebrafish (19). Baseline gene expression of RNA samples extracted from whole zebrafish embryos at 18 different developmental stages from 1 cell to 5 days post-fertilisation was based on data derived from White et al. (20). Transcripts per million (TPM) were calculated from the raw counts by Integrated RNA-Seq Analysis Pipeline (iRAP). Subsequently, they were averaged for each set of technical replicates, and then quantile normalized within each set of biological replicates using Linear Models for Microarray Data (limma). Finally, they were averaged for all biological replicates (20).

### 2.5 SNAD1 gene expression pattern in different tissues or anatomical sites of zebrafish

We compared the reference of normal gene expression patterns of si:dkey-96g2.1 (ENSDARG00000097725), a putative SNAD1 homolog, among different zebrafish tissues, including: liver, mesonephors, head kidney, spleen, bone element, intestine, granulocyte, tail, swim bladder, larva, zone of skin, muscle tissue, structure with developmental contribution from neural crest and head using the Bgee database (https://www.bgee.org/) (21), which is based exclusively on curated healthy wild-type expression data (e.g., no gene knock-out, no treatment, no disease) and is annotated to the Uberon ontology of anatomy.

### 2.6 Identification of SNAD1 sequences among “uncharacterized proteins” in published papers

Utilizing PubMed and Google web browser we searched previously published articles in the field of immunology, using the following keywords: “fish immunology”, ,,fish infections”, ,,fish immunization”, “fish pathogens”, “fish viruses”, “acclimation to cold in fish” and “stress response in fish” in terms of presence of “uncharacterized proteins” and transcripts, which showed significant changes. Using the NCBI protein and genomic databases and The Zebrafish Information Network (*ZFIN*), providing genetic and genomic data for the zebrafish (*Danio rerio*), along with the Basic Local Alignment Search Tool (BLAST) we checked all of found “uncharacterized proteins” sequences and/or “uncharacterized proteins” accession numbers, as well as, to date “uncharacterized” genomic sequences of SNAD1 (gene si:dkey-96g2.1; ENSDARG00000097725) for the presence of SNAD1. Results from NCBI protein database about the similarity to SNAD1 were based on information from Conserved Domain Databases (CDD), a protein annotation resource that consists of a collection of well-annotated multiple sequence alignment models for ancient domains and full-length proteins.

### 2.7 Experimental validation of SNAD1 in carp subjected to environmental and pathogenic stressors

#### 2.7.1 Confirmation of Cap31 homology with SNAD1 after cloning and sequencing of Cap31

In our previous study (7) we performed cloning and sequencing of carp SNAD1 (which was named as Cap31 at that time) (detailed information about experimental samples and procedures are available at ref (7). Reinforcement of the identification of Cap31 as SNAD1 was performed using the Motif Finder program (available at https://www.genome.jp/tools/motif/) (22) and a multiple sequence alignment with Clustal Omega program (available at https://www.ebi.ac.uk/jdispatcher/msa/clustalo) (23).

#### 2.7.2 Investigating SNAD1 response to temperature changes and bacterial and viral infection

The samples collected during earlier published experimental infections or temperature adaptations were used. Carps from the koi strain were experimentally infected with viruses by cohabitation with carriers of carp edema virus (CEV) as described here (24). For koi herpesvirus (KHV) PS carp strain was used and infection was performed by bath as described here (25). In case of both viral infection liver tissues were used. Bacterial infection with *Aeromonas salmonicida* was performed by intraperitoneal injection of R3xR8 cross as described here (26), liver, spleen, kidney and testis were used. Infections were performed at specific temperatures optimal for each pathogen: 18°C for CEV, 23°C for KHV and 25°C for *A. salmonicida*. Additionally samples (liver, testis, spermatic duct) from the temperature acclimation experiment leading to the first experimental description of SNAD1 is fish were used. Fish from G strain were acclimated to 10°C or 30°C for 5 weeks as described here (7). Additionally tissue library from brain, liver, kidney, head kidney, spleen, skin, gills, intestine, testis and heart obtained from R20xR8 cross were used as described here (27).

The details about animal experiments was published in references above. Experimental procedures were performed in accordance with national and international regulations for experimentation with animals with approvals from the Animal Experiments Committee in Olsztyn, Poland (no. 93/2011), the Local Ethical Commission in Krakow, Poland with allowance (no. 49/2020), the Local Ethical Committee in Lublin, Poland (no. 32/2020) and the Lower Saxony State Office for Consumer Protection and Food Safety (LAVES), Oldenburg, Germany (no. 33.19–425 2-04-16/2144).

#### 2.7.3 Quantitative-PCR analysis of SNAD1 gene expression in carp tissue under various infections and temperature changes

Total RNA was isolated from the tissues using the TRI reagent (Sigma) and transcribed to cDNA using 100 U Maxima Reverse Transcriptase (Thermo Fisher Scientific) as described earlier (7, 24–26). cDNA samples were diluted 1:40 with nuclease-free water (Thermo Fisher Scientific) before qPCR analysis. Plasmid-based quantification using SYBR Green intercalating dye qPCR was performed on duplicate samples, using Maxima SYBR Green/ROX qPCR Master Mix (Thermo Fisher Scientific). The sequences of the primers are listed in **Supplementary Table 1**. The expression of analyzed genes was assessed relative to 40S ribosomal protein S11 (40S) reference gene. The results are presented as fold changes or normalized copies per 100 000 copies of reference gene. For the presentation of the results, a new gene nomenclature was used to differentiate between multiple genes of common carp by including the last three numbers of the LOC in the gene name (**Supplementary Table 1**).

#### 2.7.4 Statistical analysis

The expression of all SNAD1 gene variants in different tissues was analysed using SigmaPlot 12.5 software (Systat Software GmbH, Germany). Prior to analysis, gene copy number values were logarithmically transformed and tested for equal variances and normal distribution. One-way ANOVA with Holm-Sidak *post hoc* test was used for comparisons of multiple experimental groups. For comparisons between two experimental groups, the Student t-test or Mann-Whitney U-test was used. Statistical significance was set at p < 0.05.

## 3 Results

### 3.1 Phylogeny of SNADs from various fish species

**Figure 3:**
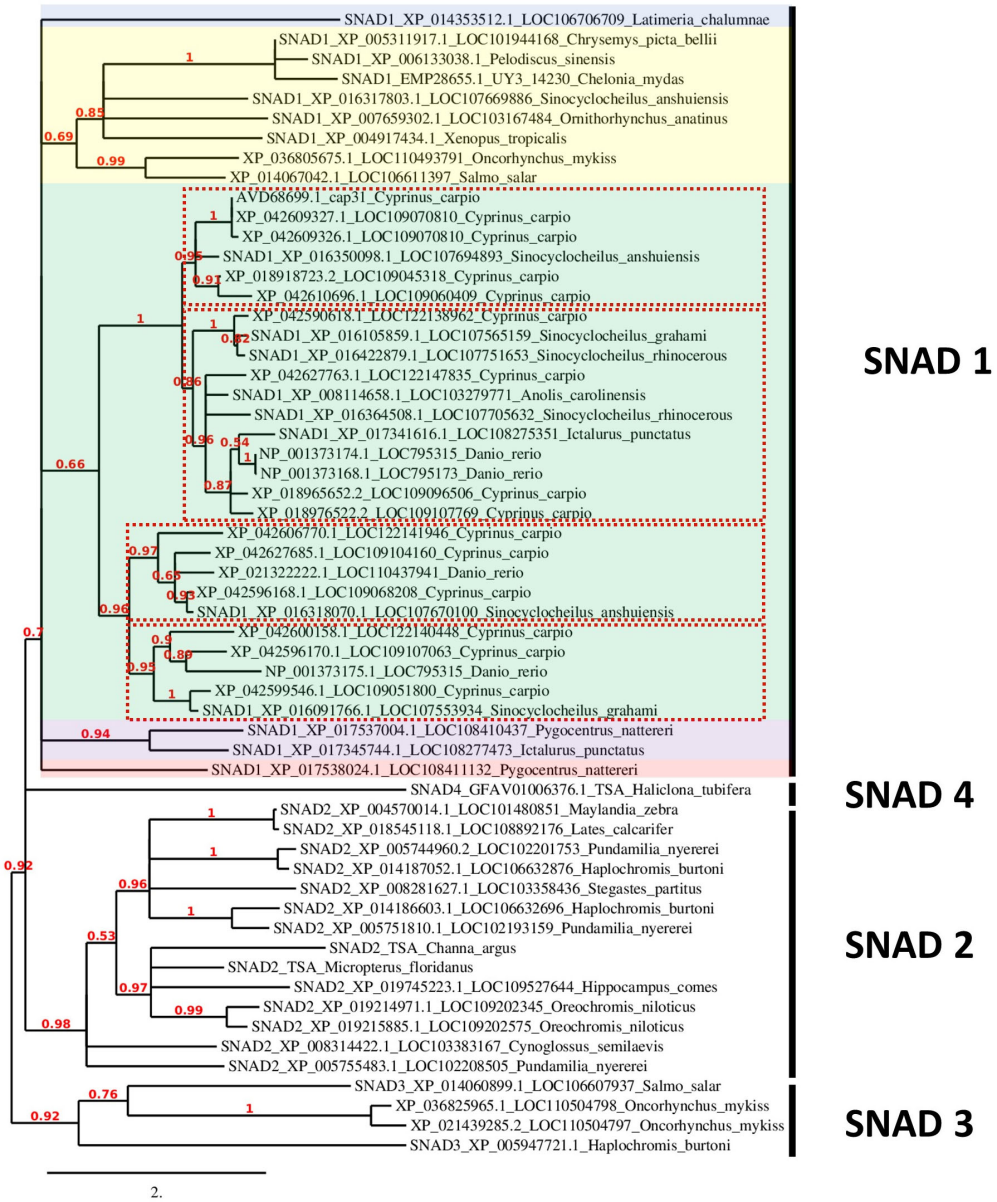
Phylogenetic analysis of SNADs from various animals. A new phylogenetic analysis of all available SNADs in common carp (*Cyprinus carpio*), zebrafish (*Danio rerio*), rainbow trout (*Oncorhynchus mykiss*) and Atlantic salmon (*Salmo salar*) compared to SNAD1, SNAD2, SNAD3 and SNAD4 members previously analysed by et al. (4) and SNAD1 and SNAD2 from fish previously analysed by Bakke et al. in 2022 (25). The previously analysed sequences are indicated with SNAD1-4 at the start of the sequence name. Furthermore, each sequence name contains the protein sequence GenBank ID and LOC number or gene name abbreviation as well as Latin name of the species. The sequences were analysed for their phylogenetic relationships to other SNAD sequences found in GenBank with the tools available at www.phylogeny.fr (7), and they were aligned with MUSCLE (8). The phylogenetic analyses (based on maximum likelihood) were performed with PhyML (9), and finally, the phylogenetic tree was rendered with TreeDyn (10). The numbers indicate the branch support value. The branch length is reflecting the number of substitutions per site. Different colour shading indicates divergent branches of SNAD1. The different clades of SNAD1 from warm-adopted fish are indicated by red dotted line rectangles.

Phylogenetic analysis using all available SNADs in common carp, zebrafish, rainbow trout, and Atlantic salmon, and earlier described SNAD1, 2, 3, and 4 proteins revealed a distinct separation between different SNADs. Furthermore, the SNAD1s show several distinct branches. Within the SNAD1, we found additional separation into four distinct clades withing the branch formed by warm-adopted fish (**Figure 3**). Interestingly, cold water-adapted salmonids from additional SNAD1 cluster which was different from SNAD1 formed by those of warm water-adapted cyprinids, cichlids, and serrasalmids (**Figure 3**). Salmonids SNADs are also present in the SNAD3 cluster.

### 3.2 *In silico* characterization of SNAD gene structure and expression patterns

Given the large number of SNAD1 sequences found in common carp, we performed an *in silico* analysis of these sequences. The SNAD1 sequences were BLAST searched against the common carp genome (ASM1834038v1 in the NCBI database). The number of exons, the genomic location (chromosome number and nucleotide region) and experimental validated expression presence and regulation type of 13 genes encoding potential SNAD1 in carp after environmental and pathogenic challenges were reported (see below 3.72 and 3.73) (**Table 1**). Sequences were also checked against EST, TSA and proteome databases, which may indicate that genes are expressed or present as protein in their host. Of the thirteen SNAD1 genes, eleven were located on a defined chromosome in the carp genome. One of the genes (LOC109045318) mapped to an undefined chromosome is expressed in the liver (EST CA967515) and present in the seminal plasma proteome and is therefore most likely functional. The mRNA of four genes LOC109051800, LOC109107769, LOC122138962, LOC109104160, LOC109070810 [also known as Cap31, ref (7)] were detected in the transcriptome shotgun analysis sequence database, LOC122138962, LOC122140448 and LOC109070810 were also present in the EST libraries. In zebrafish, three SNAD1 genes have been reported and BLAST analysis against the GRCz11 reference genome showed that they are all located on chromosome 15 and have three exons. Searches of the TSA and EST libraries showed that zebrafish genes are widely expressed in various tissues (**Table 1**). The same is true for the expression of the single SNAD1 genes in rainbow trout and Atlantic salmon, which have three and four exons respectively (**Table 1**).

**Table 1:** Results of in silico analysis of gene structure.

Species	LOC	Protein	mRNA	No. Exons	Chromosome	TSA	EST	Proteome	Experimental validated mRNA expression	Experimental validated expression regulation type		
										Temperature	Bacterial infection	Viral infection
*Cyprinus* *carpio*	LOC109045318	XP_018918723	XM_019063178	2	Unkn (NW_024879241) 645235-646428	–	Liver (CA967515)	Seminal plasma (+)	Liver (+)			
*Cyprinus* *carpio*	LOC109051800	XP_042599546	XM_042743612	2	B18 (NC_056614) 19232994* -19231972	Skin/scale (GFWU01012140)	–	–	Liver (+/-)			
*Cyprinus* *carpio*	LOC109060409	XP_042610696	XM_042754762	2	Unkn (NW_024879241) 1279916 -1287517	–	–	–	Liver (-)			
*Cyprinus* *carpio*	LOC109096506	XP_018965652	XM_019110107	3	B9 (NC_056605) 1077174-1075311	–	–	Seminal plasma (+)	Liver (+)		**↑**	**↑**
*Cyprinus* *carpio*	LOC109107063	XP_042596170	XM_042740236	3	B15 (NC_056611) 28145330-28147608	–	–	–	Liver (+)	**↑**		**↑**
*Cyprinus* *carpio*	LOC109107769	XP_018976522	XM_019120977	3	B15 (NC_056611) 28161714- 28164321	Skin/scale (GFWU01037700)	–	–	Liver (+)	**↑**	**↑**	
*Cyprinus* *carpio*	LOC122138962	XP_042590618	XM_042734684	3	A3 (NC_056574) 6521949-6525096	Skin/scale (GFWU01039137)	Mix# JZ506855	–	Liver (+)			**↑**
*Cyprinus* *carpio*	LOC122141946	XP_042606770	XM_042750836	3	B23 (NC_056619) 4518789-4519769	–	–	–	Liver (+)			**↑**
*Cyprinus* *carpio*	LOC122147835	XP_042627763	XM_042771829	3	A15 (NC_056586) 25314558-25315893	–	–	Seminal plasma (+)	Liver (+)	**↑**	**↑**	
*Cyprinus* *carpio*	LOC109070810§	XP_042609326	XM_042753392	3	B25 (NC_056621) 19582969-19584315	Skin/scale (GFWU01012401)	Liver (CA964173)	Seminal plasma (+) Blood (+)	Liver (+)	**↑**	↓	↓
									Kidney (+)			
									Spleen (+)			
									Testis (+)		↓	
									Spermatic duct (+)			
									Brain (+)			
									Gills (+)			
									Intestine (+)			
									Heart (+)			
									Spleen (+)			
									Spermatic duct (+)			
*Cyprinus* *carpio*	LOC122140448	XP_042600158	XM_042744224	3	B18 (NC_056614) 19245032-19241247	–	Testis (DW721040)	–	Liver (+)	↓		↓
*Cyprinus* *carpio*	LOC109068208	XP_042596168	XM_042740234	3	B15 (NC_056611) 28118411-28120031	–	–	–	Liver (+)		**↑**	**↑**
*Cyprinus* *carpio*	LOC109104160	XP_042627685	XM_042771751	3	A15 (NC_056586) 25309210-25312825	Skin/scale (GFWU01035844)	–	–	Liver (+)			
*Danio rerio*	si:dkey-96g2.1	NP_001373174	NM_001386245	3	15 (NC_007126) 42048022-42042138	Kidney/intestine/ gills/spleen (GDQQ01003258)	Multiple sequences	n.a.	n.a.	n.a.	n.a.	n.a.
*Danio rerio*	LOC795173	NP_001373168	NM_001386239	3	15 (NC_007126) 42086236-42079864	Multiple tissues (GFIL01024918)	Multiple sequences	n.a.	n.a.	n.a.	n.a.	n.a.
*Danio rerio*	LOC110437941	XP_021322222	XM_021466547	3	15 (NC_007126) 42059140-42053981	Multiple tissues (GFIL01029548)	Brain (CF550236)$	n.a.	n.a.	n.a.	n.a.	n.a.
*Oncorhynchus mykiss*	LOC110493791	XP_036805675	XM_036949780	3	17 (NC_035093) 20153206-20156778	Multiple tissues (GFIN01025218)	Multiple tissues (BX911094)	n.a.	n.a.	n.a.	n.a.	n.a.
*Salmo salar*	LOC106611397	XP_014067042	XM_014211567	4	ssa09 (NC_027308) 44034498-44039525	Multiple tissues (GGAQ01011846)	Gut (CB504138)	n.a.	n.a.	n.a.	n.a.	n.a.

The number of exons and the chromosome encoding the SNAD1 genes are given. The existence of mRNA transcripts was checked in expressed sequence tags (EST) and transcriptome shotgun assembly (TSA) databases, and matching accession numbers are indicated. The presence of proteins was checked in in-house common carp proteomes data bases of blood and sperm plasma. Experimental validated expression presence and regulation type of 13 genes encoding potential SNAD1 in carp after environmental and pathogenic challenges are given.

* partial sequence

# cDNA from brain, gill, heart, blood, head-kidney, kidney, liver, and gonad

§ - cap31 gene (MG457251)

$ - 91% similarity

n.a. – not analysed

"-" indicates no data or not applicable

**↑ ** - indicates upregulation

↓ - indicates downregulation

Unkn - unknown.

### 3.3 Predictions of SNAD1 secondary and tertiary structure in the background of AADs structure

**Figure 4:**
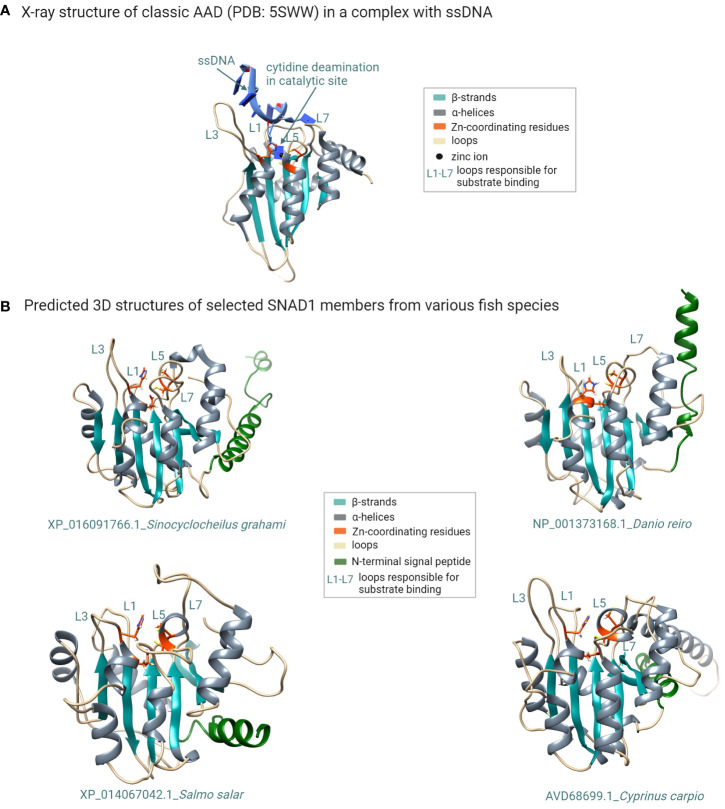
Structural modeling of SNAD1 members reveals their common structural features shared with classic AADs. **(A)** X-ray structure of the classic AAD enzyme (APOBEC3A; PDB: 5SWW) in complex with a ssDNA substrate. The typical fold of classic AADs consists of five β strands (cyan) and six α helices (grey). The two conserved Cys residues and one His residue that coordinate the zinc atom in the catalytic centre are shown in orange. Additionally glutamate (also shown in orange) acts as a proton donor. The loops surrounding the catalytic site (believed to be responsible for substrate recognition) are labelled L1, L3, L5, and L7. **(B)** Multiple sequence alignment of selected SNAD1 proteins with the indicated conserved residues (orange arrows) forming the catalytic centre characteristic of zinc-dependent deaminases. **(B)** 3D structural models (generated using the RoseTTAFold method) of selected SNAD1 members from various fish species. The zinc-coordinating motif in the predicted catalytic centre (orange) is located at a position analogous to that in classic deaminases, and the catalytic centre is surrounded by the L1, L3, L5, and L7 counterparts of those in classic AADs. The signal peptide is coloured green. (Figure created with http://biorender.com).

To check a structural similarity between predicted SNAD1 members, we performed primary amino acid sequence alignment of all SNAD1s identified in our phylogenetic analysis. To the comparison, we added also a well-characterized AAD representative (APOBEC3A) for which 3D structure with good resolution is available (PDB: 5SWW). The comparison of the primary structures revealed that almost all of the analyzed sequences (except XP_042609327.1 and XP_014353512.1) contained highly conserved zinc-coordinating motif, characteristic for zinc-dependent deaminases (1) in which two conserved Cys residues, one His residue and a water molecule coordinate a zinc ion, and glutamate (within HxE motif) acts as a proton donor (**Supplementary Figure 1**). The context of the two conserved Cys residues was marginally different for SNAD1s and AADs (CVxxC versus Cx_2-4_C for SNAD1s and APOBECs, respectively). However, this did not influence the common core fold of the catalytic center as evidenced by the location of conserved residues in the primary structure (shown in red in **Supplementary Figure 1**; similarity score 0.7) and the similarity of 2D structures (presented above the text in **Supplementary Figure 1**) for this catalytic region. To check the similarity between 3D structures of SNAD1s and 3D structure of the AAD representative, we performed *ab initio* modeling using the RoseTTAFold method. For the modeling, we selected 12 sequences, each corresponding to a divergent branch of SNAD1, marked by different colour shading in **Figure 3**. Representative examples of the generated 3D structures are presented in **Figure 4** and the whole set of predictions is included in **Supplementary Figure 3**. We also performed superposition of the predicted 3D structures of SNAD1s from warm-water-adapted (XP_021322222.1 *Danio rerio*) and cold-water-adapted (XP_014067042.1 *Salmo salar*) fish (**Supplementary Figure 2A**), as well superposition of 3D structure of SNAD1 representative (XP_021322222.1 *Danio rerio*) and AAD representative (APOBEC3A, PDB: 5SWW) (**Supplementary Figure 2B**). In SNAD1 3D structures we identified the common core fold consisting of one β sheet surrounded by α helices. The topologic similarity between warm-water-adapted and cold-water-adapted SNAD1s (expressed as template modeling score) was high reaching 0.65. As expected, the template modeling score was slightly lower between AAD and SNAD1 reaching 0.4. The template modeling score measure ranges between 0 and 1, where 1 indicates a perfect match and 0 is no match between structures. Is accepted that scores < 0.2 indicate unrelated proteins while > 0.5 indicate the same protein fold. Moreover, we showed that the zinc-coordinating motif in the predicted catalytic centre is located at a position analogous to that in classic deaminases (**Figure 4**; **Supplementary Figure 3**), and the catalytic centre is surrounded by structures equivalent to L1, L3, L5, and L7 in classic AADs (**Figure 4**; **Supplementary Figure 3**). For 26 out of 38 analyzed SNAD1 proteins, we identified N-terminal signal peptides suggesting their secretory nature. The signal peptides differed significantly in both sequence and length (from 17 to 33 amino acids, on average 24). Interestingly, for six analyzed SNAD1 proteins (XP_016091766.1; XP_042627763.1; XP_016422879.1; XP_016105859.1; XP_042609326.1; XP_042609327.1) the N-terminal region formed a membrane-anchored structure. For almost all of the analyzed SNAD1 proteins (except XP_042609327.1 and NP_001373175.1), we identified a C-terminal cluster of three Cys residues, which according to the published data (4) seem to be characteristic of SNADs, and likely form disulfide bonds stabilizing two most C-terminal part of the enzyme backbone.

### 3.4 SNAD1 gene expression at different developmental stages in zebrafish

**Figure 5:**
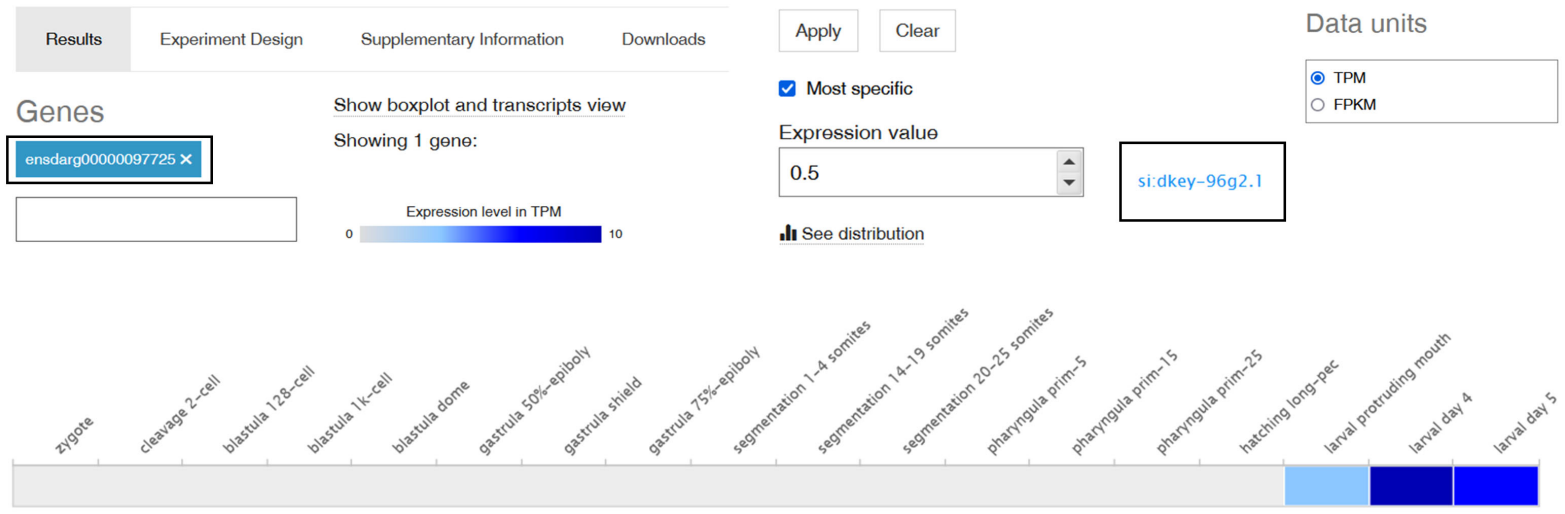
Expression of the si:dkey-96g2.1 gene (encoding a putative SNAD1 homolog) in different developmental stages in zebrafish. A screenshot from Expression Atlas presenting a si:dkey-96g2.1 gene transcript expression levels as TPM (transcript per million) across all 18 developmental stages of zebrafish. The grey box represents expression levels below the cutoff (0.5 TPM), the light blue box indicates low expression levels (between 0.5 and 10 TPM), and the medium blue box signifies medium expression levels (between 11 and 1000 TPM).

Among all of the analysed SNAD1 sequences, the only zebrafish gene available in the databases assigned as an “uncharacterized protein” similar to SNAD1 was si:dkey-96g2.1 (ENSDARG00000097725). Therefore, using an Expression Atlas (http://www.ebi.ac.uk/gxa) tool (19), we found that SNAD1 transcript was first transcribed at the end of embryogenesis (protruding mouth stage). In this stage, a low gene expression level (3 transcripts per million, TPM) was observed. In subsequent developmental stages, gene expression levels on larval days 4 and 5 were intermediate (10 TPM) and low (5 TPM), respectively (**Figure 5**).

### 3.5 SNAD1 gene expression pattern in different tissues or anatomical sites of zebrafish

**Figure 6:**
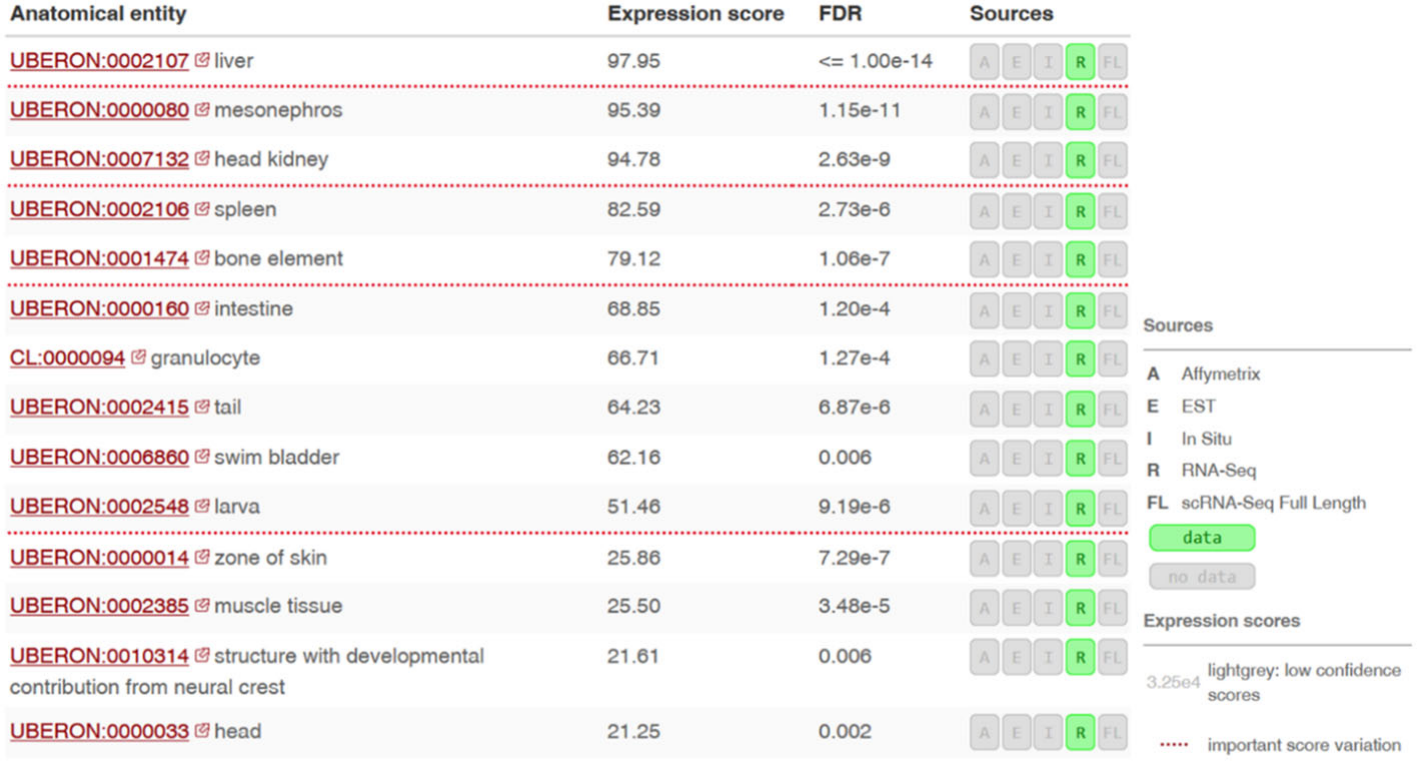
Expression levels of the si:dkey-96g2.1 gene (encoding a putative SNAD1 homolog) in different tissues or anatomical sites of zebrafish. RNA-seq data ilustrating the si:dkey-96g2.1 gene expression across various tissues and anatomical sites in zebrafish using the Bgee tool. Expression scores are based on the rank of a gene in a condition according to its expression levels (nonparametric statistics), normalized using the minimum and maximum rank of the species. Values of Expression scores are between 0 and 100. Low score means that the gene is lowly expressed in the condition compared to other genes. Scores are normalized and comparable across genes, conditions and species.

Using the Bgee database (21), we found the highest gene expression in the liver, mesonephros (posterior kidney), pronephros (head kidney) and spleen (**Figure 6**).

### 3.6 Identification of SNAD1 sequences among uncharacterized proteins in published papers

**Figure 7:**
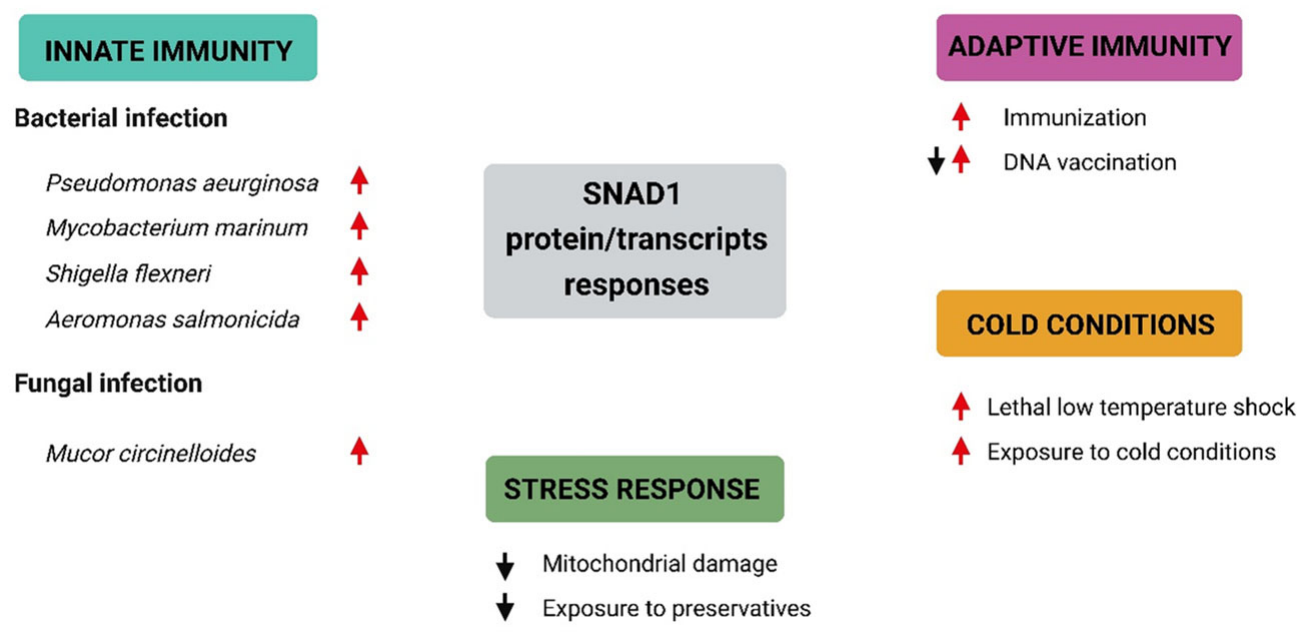
Selected SNAD1 protein/transcript responses under different conditions based on data included in **Table 2**. Summary of current knowledge regarding SNAD1 responses under different conditions. Red arrows represent upregulation of SNAD1 proteins/transcripts, while black arrows indicate downregulation of SNAD1 proteins/transcripts.

We found SNAD1 sequences that were described as “uncharacterized proteins” and/or transcripts in 19 studies (key results, with emphasis on changes in SNAD1, are summarized in **Table 2**; **Figure 7**). The results of our search indicate the involvement of SNAD1 in various immunological processes, including the immune response due to bacterial infection or immunization, and its involvement in interactions with the intestinal microbiota. Moreover, we identified a relationship between SNAD1 and temperature acclimation, as well as its association with environmental pollution. Additionally, we demonstrated that SNAD1 levels are influenced by sex, exhibiting higher abundance in females, and that it plays a role in proper pancreas development (40, 41, 45).

**Table 2:** Results from an analysis of previously published papers reporting the detection of uncharacterized proteins/transcripts similar to SNAD1.

Area of research	Main subject	Main conclusions regarding SNAD1	Identified proteins/transcripts similar to SNAD1	Experimental material	Species	Reference
Immunology	Response following immunization	Possible marker protein for different phases of the immune response	XP_021423950.1 XP_021423951.1	Plasma	Rainbow trout [*Oncorhynchus mykiss*]	(28)
Immunology	Response to bacterial infection	Overexpressed	XP_021423951.1 XP_014067041.1	Liver	Rainbow trout [*Oncorhynchus mykiss*]	(29)
Immunology	Enteroendocrine cell response to bacterial infection	Overexpressed	ENSDARG00000097725	Larvae	Zebrafish [*Danio rerio*]	(30)
Immunology	Commensal microbes affect intestinal physiology	Marker of progenitor-like cells	ENSDARG00000097725	Intestine	Zebrafish [*Danio rerio*]	(31)
Immunology	Intestinal mucosal immunity affected by DNA vaccination	Down- and upregulation of KTF82305.1 and KTF82307.1 protein expression, respectively in response to immunization	KTF82305.1 KTF82307.1	Intestine	*Grass carp* [*Ctenopharyngodon idella*]	(32)
Immunology	Spleen development	Significantly modulated expression during ageing	NP_001373174	Spleen	*Grass carp* [*Ctenopharyngodon idella*]	(33)
Immunology	Host aryl hydrocarbon receptor (AhR) signalling during *P. aeruginosa* infection	Overexpressed 24 h post-infection	si:dkey-96g2.1	Larvae	Zebrafish [*Danio rerio*]	(34)
Immunology	Response to infection with a cystic fibrosis-associated isolate of *P. aeruginosa*	Overexpressed	si:dkey-96g2.1	Larvae	Zebrafish [*Danio rerio*]	(35)
Immunology	Response to *M. marinum* infection in galanin+/+ larvae	Overexpressed	si:dkey-96g2.1	Larvae	Zebrafish [*Danio rerio*]	(36)
Immunology	Response to *S. flexneri* infection	Overexpressed	ENSDARG00000097725	Larvae	Zebrafish [*Danio rerio*]	(37)
Immunology	Response to *M. circinelloides* infection	Overexpressed	ENSDARG00000097725	Kidney	Zebrafish [*Danio rerio*]	(38)
Acclimation to cold/ Immunology	Responses associated with acclimation to cold and warm temperatures	Upregulation upon adaptation to cold temperature	KTG44393.1	Plasma	Carp [*Cyprinus carpio L*]	(7)
Acclimation to cold/ Immunology	Cold-induced damage to zebrafish larvae	Differential regulation of gene expression in relation to temperature	si:dkey-96g2.1	Larvae	Zebrafish [*Danio rerio*]	(39)
Sex dimorphism	Sex-based proteome of the heart	Higher expression in females than in males	X1WGQ3	Heart	Zebrafish [*Danio rerio*]	(40)
Sex dimorphism	Sex-based proteome of the plasma	Higher expression in females than in males	XP_005157870	Plasma	Zebrafish [*Danio rerio*]	(41)
Environmental pollution/ toxicology	Effects 4-nonylphenol, triclosan, and triclocarbanan exposure	Changes in expression following exposure to triclocarbanan	si:dkey-96g2.1	Larvae	Zebrafish [*Danio rerio*]	(42)
Environmental pollution/ toxicology	Effects of amisulbrom (AML) and isoflucypram (ISO) on zebrafish embryogenesis	Differential regulation of gene expression following exposure to AML and ISO	ENSDARG00000097725	Fertilized embryos	Zebrafish [*Danio rerio*]	(43)
Blood coagulation	Tissue Factor Pathway Inhibitor (TFPI) regulation	Higher expression in wild-type fish than in knockout homozygotes	X1WGQ3	Larvae	Zebrafish [*Danio rerio*]	(44)
Development	Pancreas development upon mutation of genes in the MIA pathway	Downregulation in mia40a mutants	si:dkey-96g2.1	Larvae	Zebrafish [*Danio rerio*]	(45)

### 3.7 Bridging *in silico* predictions with direct evidence: presence of SNAD1 in carp, exploring SNAD1 in carp’s adaptation to temperature and infection

#### 3.7.1 Confirming SNAD1’s presence in carp: cloning and sequencing

**Figure 8:**
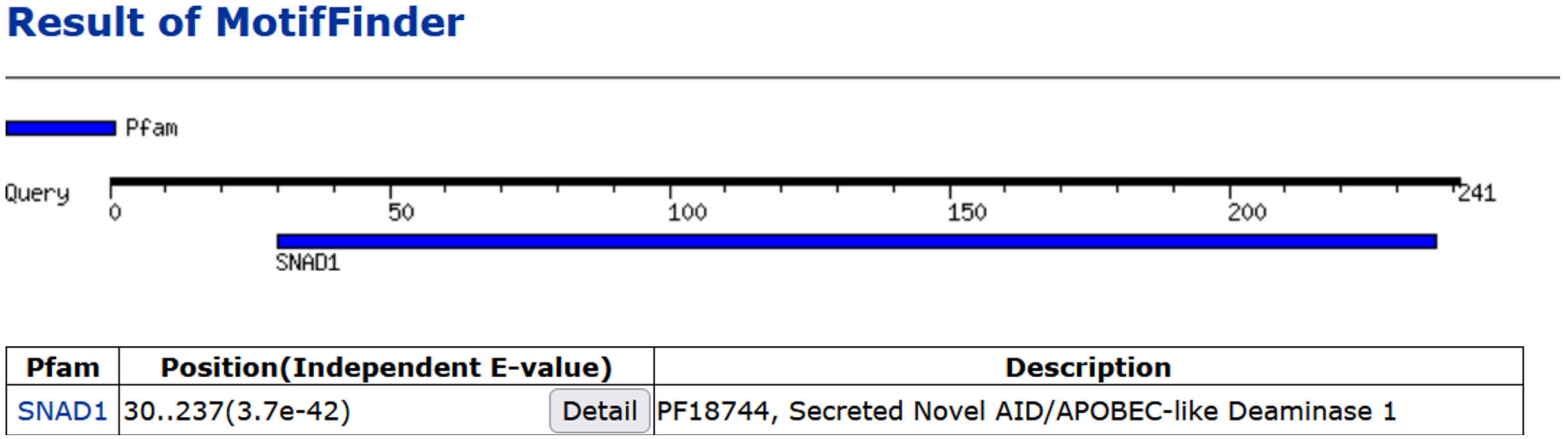
Homology of Cap31 (AVD68699.1) with SNAD1 protein indicated with the use of Motif Finder tool.

In 2018 in our study we provided, for the first time, experimental direct evidence confirming the presence of SNAD1 in carp (7). We conducted cloning and sequencing of SNAD1 (which was named as Cap31 at that time), measured its expression in tissues and detected SNAD1 protein through 2D gel electrophoresis. The sequence was deposited in the GenBank under the accession number AVD68699.1. Using the Motif Finder program for analysis, this sequence was conclusively identified as SNAD1 (**Figure 8**). Furthermore, a multiple sequence alignment, comparing the cloned Cap31 sequence with known SNAD1 sequences further reinforced the identification of Cap31 as SNAD1 (results available at ref, (7)). Multiple alignment of cloned Cap31 protein clearly identified it as SNAD1 (7).

#### 3.7.2 Preliminary findings on expression of Cap31 (snad1_810)

**Figure 9:**
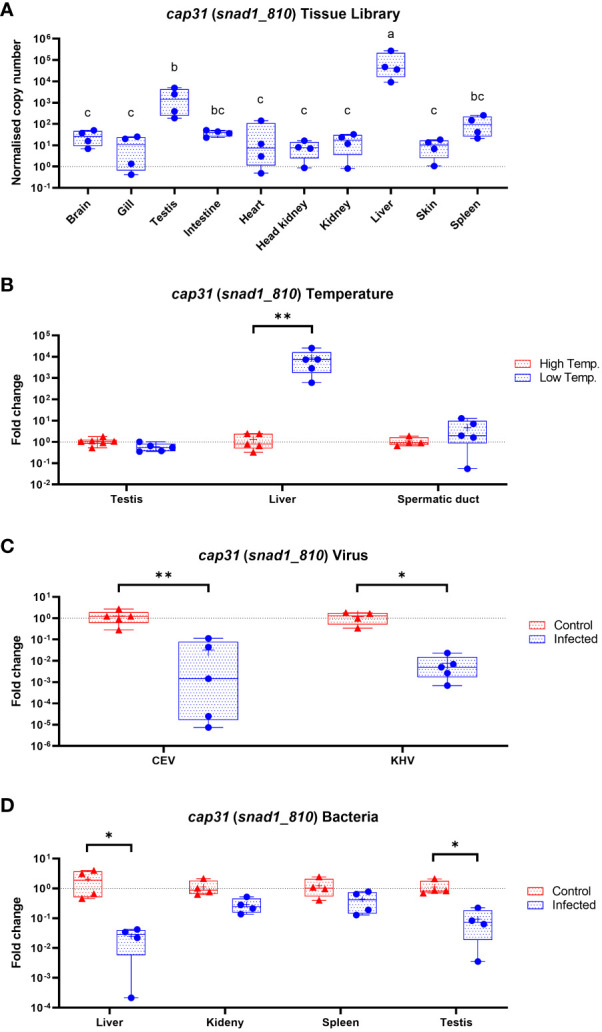
Expression of Cap31 (snad1_810) in carp tissue library (n = 4-6) **(A)**, in carp tissues (testis, liver and spermatic duct) acclimated to different temperatures **(B)**, in liver after infection of carp with viruses (CEV, KHV) **(C)** and in liver, kidney, spleen and testis during bacteria infection (*Aeromonas salmonicida*) **(D)**. Tissue library results are shown as normalized copies while results from infections and temperature acclimation as fold changes towards the control. Carp were maintained at 18°C for CEV, 23°C for KHV and 25°C for *A. salmonicida* and 10°C/30°C for temperature adaptation experiments. Differences in-between tissues in tissue library are indicated with different letters for P < 0.05. Differences between control and infected individuals or acclimated to different temperatures individuals are indicated as * and for P < 0.05, ** for P < 0.01.

We used Cap31 gene (snad1_810) as a prototypical member of SNAD1 encoding genes in carp. We measured the expression of the gene and concluded that the biggest expression is present in liver followed by testis, spleen, intestine and brain. Interestingly, the Cap31 was regulated only in liver and not in testis and spermatic duct during temperature adaptation. During infection of carp with viruses (CEV and KHV) and bacteria (*Aeromonas salmonicida*) the Cap31 expression was negatively affected in livers (**Figure 9**). Additionally, we tested testis, spleen and kidney during the bacteria infections and expression Cap31 was downregulated in testis.

#### 3.7.3 Multiple SNAD1 variants in carp: a new avenue for research

**Figure 10:**
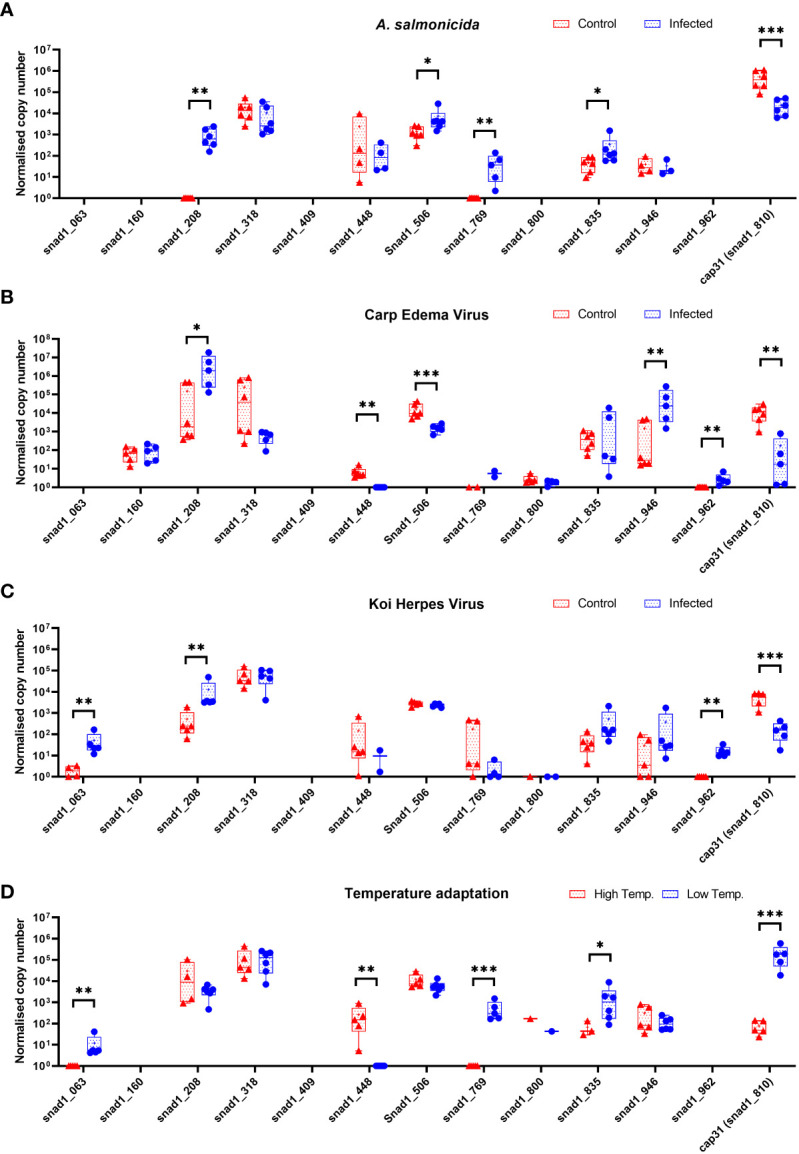
Expression of genes encoding for potential SNAD1 in carp liver (n = 4-6) after infection of carps with bacteria (*Aeromonas salmonicida*) **(A)** and viruses (CEV, KHV) **(B, C)** and themperature adaptation **(D)**. Carps were maintained at 18°C for CEV, 23°C for KHV and 25°C for *A. salmonicida* and 10°C/30°C for temperature adaptation experiments. Differences between control (CTRL) and infected individuals or acclimated to different temperatures individuals are indicated as * and for P < 0.05, ** for P < 0.01, *** for P < 0.001.

The initial experiments focused on just one of several SNAD1 variants present in carp (**Figure 3**). Therefore, we performed the expression studies of other genes encoding for potential SNAD1 proteins only in liver samples which were indicated to the most affected in case of Cap31 (**Figure 10**). Distinct patterns of expression emerged under various infections and temperature changes. Genes snad1_063, snad1_448, snad1_769, and snad1_810_cap31 exhibited changes in expression levels in response to both infection and elevated temperatures. On the other hand, snad1_160 showed no significant regulation, indicating a potential baseline function of this variant unaffected by the tested stressors or a highly specific regulatory mechanism not triggered under the study’s conditions. Similarly, variants snad1_316, snad1_835, and snad1_946 were also found to have no regulation or high expression levels without specific regulation, suggesting roles in essential cellular processes not influenced by infection or temperature changes. The response to infection alone was observed in snad1_208, snad1_506, and notably, snad1_962, where the expression was specifically upregulated by viral infection. This indicates a potential role of these variants in the carp’s antiviral response, suggesting that snad1 may be involved in the recognition or inhibition of viral pathogens. Noteworthy is the absence of expression observed in snad1_409 and the near absence in snad1_800, suggesting the need for further primer optimisation or highly context-dependent expression patterns that may be silenced under certain conditions/or in certain tissues, or it may also be an indicator of dysfunction of these sequences as genes.

## 4 Discussion

The inspiration for this research was derived from the results of our previous study (7), in which we identified an “uncharacterized protein” named Cap31 showing a significant response to cold acclimation, which was later identified as SNAD1. Remarkable changes in expression, such as a 13-fold increase in Cap31 protein abundance upon adaptation to cold temperature and a 1000-fold higher expression of Cap31 mRNA in the livers of carp acclimated to cold temperature compared to those at warm temperature (7), guided us to investigate SNAD1 through available scientific methodologies.

### 4.1 SNADs in fish can be divided into cold and warm water-adapted fish species

We made a phylogenetic analysis of all available SNADs in common carp, zebrafish and rainbow trout in comparison to SNAD1, SNAD2, SNAD3 and SNAD4 members previously analysed by Kirshnan et al. (4) and SNAD1 and SNAD2 from fish previously examined by Bakke et al. (46). The sequences were analysed for their phylogenetic relationships to other SNAD sequences found in GenBank. Our analyses revealed a distinct separation between SNADs of cold water-adapted salmonids from those of warm water-adapted cyprinids, cichlids and serrasalmids. Within these groups, we identified additional division into two to three separate clades (**Figure 3**). These shows that the SNADs are far more heterogenic family of proteins in fish. It should be noted, that the observed separation between cold and warm water-adapted fish species might be associated with the presence of overexpressed levels of SNAD1 as a response to cold exposure in fish (as discussed below). Additionally, due to unique cold adaptations of arctic fish, we checked them for the presence of SNAD1 motifs. Using the Motif Finer tool, we found homology of uncharacterized protein LOC117450837 (NCBI Reference Sequence: XP_033944723.1) from South Georgia icefish (*Pseudochaenichthys georgianus)* with SNAD1 (unpublished data). Further studies are needed to explore the potential role of SNAD1 in exceptional mechanisms of cold adaptation in arctic fish.

### 4.2 SNAD1 shares similar core structural features with AADs and possibly zinc-dependent cytidine deaminase activity

Classic AADs share similar core structural features with other zinc-dependent deaminases (**Figure 4A**). Their catalytic centre is characterized by the canonical structural motif HxEx25-30PCx2-4C, in which two conserved Cys residues, one His residue and a water molecule coordinate a zinc ion (47). The motif is located within one or two deaminase domains, whose typical fold comprises five β strands that form the enzyme backbone, which is surrounded by six α helices. The mechanism of enzymatic deamination is conserved within the whole superfamily of zinc-dependent deaminases and consists of nucleophilic attack at position C4 of the cytidine ring (or at position A6 of the adenine ring) by the activated water molecule, coordinated by the zinc ion and the conserved glutamate (which acts as a proton donor) (1). In support of this mechanism, mutation of glutamate or any of the zinc-coordinating residues results in a loss of enzymatic activity (48). In the structure of classic AADs, the conserved catalytic centre is surrounded by loops designated L1, L3, L5, and L7. Variability in loop length, amino acid composition, plasticity, and dynamics is believed to be critical for substrate sequence specificity (2, 49). In 3D structure models generated *ab initio* for selected SNAD1 members we identified the common core fold consisting of one β sheet surrounded by α helices (see **Figure 4**; **Supplementary Figure 3**) The zinc-coordinating motif in the predicted catalytic centres located at a position analogous to that in classic deaminases (see **Figure 4**; **Supplementary Figures 1**, **3**). Moreover, the catalytic centre was surrounded by structures analogous to L1, L3, L5, and L7, which correspond to those found in classical AADs.

Based on the common structural features of AADs and SNADs, Krishnan et al. proposed that SNADs play a role as cytosine deaminases of single-stranded nucleic acids (4). Our phylogenetic analysis supports this hypothesis, as the vast majority of sequences included in our analysis contained the expected highly conserved zinc-coordinating motif (**Supplementary Figure 1**), supporting the classification of these proteins into the zinc-dependent deaminase superfamily.

### 4.3 SNAD1 members are characterized by the presence of N-terminal signal peptides suggesting their secreted nature

We identified common structural features of SNAD1 members and investigate their potential structural variations in various fish species. As expected, in the vast majority of the analysed proteins (26 out of 38), we identified N-terminal signal peptides, suggesting that these proteins might be secreted. Among proteins, the signal peptides differ significantly in both sequence and length (from 17 to 33 amino acids, on average 24). The high diversity of this region may indicate different secretory pathways for different SNAD1 proteins. Interestingly, in the case of six SNAD1 members (XP_016091766.1; XP_042627763.1; XP_016422879.1; XP_016105859.1; XP_042609326.1; XP_042609327.1), in our predictions, the N-terminal region formed a membrane-anchored structure.

### 4.4 SNAD1 show great diversity in terms of sequence and length between fish species

For almost all of the analysed SNAD1 proteins (except XP_042609327.1 and NP_001373175.1) we identified the presence of a C-terminal cluster of three Cys residues, which are characteristic of SNADs (4). They likely form disulfide bonds stabilizing the C-terminal enzyme backbone. All the analysed proteins are also characterized by the presence of loops L1, L3, L5, and L7 surrounding the catalytic centre which corresponds to analogous found in classical AAD. Considering that these loops are likely responsible for substrate recognition (2, 4, 49) and both their length and sequence vary widely among closely related SNADs [see **Figure 4**; **Supplementary Figure 3**, and ref (**4**)], one could hypothesize that SNAD1 proteins recognize different targets or that a given type of target is structurally variable (as would be expected in the case of RNA substrates). The high flexibility of active-site loops may suggest additional degrees of freedom of a substrate in the catalytic pocket (50). Taking into account also secreted nature of SNAD1s we hypothesized RNA as their most likely substrate. Moreover, it is also possible that the variable length of these loops indicates different oligomerization statuses of SNAD1 members. The latter is rationalized by the proven involvement of loop 3 in the dimerization of tRNA adenosine deaminase (TadA) enzyme (from which AAD family members are believed to originate) (51).

In summary, the conservation of structures among SNAD1 members from different fish species supports the classification of these proteins into a common subfamily. The conservation is additionally supported by the relatively high template modeling score between 3D structures of SNAD1 members from cold- and warm-water-adapted fish (see **Supplementary Figure 3**). All analysed SNAD1 proteins exhibit structural features of zinc-dependent deaminases, including zinc-coordinating catalytic centres and similar backbones. The high variability of SNAD1 structures in the region of signal peptides and loops surrounding the catalytic centre suggests possible specialization of these enzymes, especially in terms of different secretory pathways and recognized nucleic acids.

### 4.5 SNAD1 transcripts are expressed in the immunological organs of zebrafish

Based on RNA-seq data, SNAD1 is first transcribed in zebrafish after hatching, which suggests its physiological importance during that period and later (**Figure 5**). This indicates that SNAD1 is not important during embryo development period. Moreover, we found the highest SNAD1 gene expression in the liver, mesonephros (posterior kidney), pronephros (head kidney) and spleen (**Figure 6**). Notably, all of these organs play a pivotal role in the fish immune system (52). The head kidney is the dominant haematopoietic organ, described as an analogue of bone marrow in higher vertebrates, and functions as the primary haematopoietic tissue in fishes, a description also applied to the spleen and mesonephros (52, 53). In teleost fish, the kidneys contain B and T lymphocytes, dendritic cells, neutrophils, macrophages, granulocytes, thrombocytes and melano-macrophage centres (MMCs). Moreover, mature naive IgM+ cells and IgD+ cells produced by lymphoid progenitors are able to migrate to the mesonephros and spleen. The spleen and head kidney are the main sources of thrombocytes involved in immune responses (e.g., phagocytosis and intracellular killing of pathogens) and express various components of the major histocompatibility complex (MHC) (52).

Due to the presence of AID, B cells and CD4+ T cells, the MMCs present in the kidney and liver are considered as a primitive germinal centres (GCs) able to trap and store antigens (52, 53). The results of constitutive mRNA expression analyses are consistent with our hypothesis that SNAD1 could be a crucial component of the fish immune defence system. This suggestion is strongly supported by the changes in SNAD1 levels during various immunological-related processes (see below).

### 4.6 Evidence supporting the involvement of SNAD1 in the fish immune response.

Despite the potentially important roles of SNADs in fish biology, SNADs have thus far escaped the attention of the majority of scientists. To date, in many publications, raw results regarding SNAD1 expression changes have been available but were not included in the general discussion due to their “uncharacterized” status. However, recently, we identified homology between these sequences and SNAD1, which allowed us to interpret obtained results and speculate about SNAD1 possible functions and its involvement in various biological processes.

We identified SNAD1 as a protein whose expression is highly modulated during immune responses. Possible roles of this protein have been suggested by several studies related to innate immunity, enhancement of innate immunity by cold conditions, stress responses and adaptive immunity in fish (**Table 2**; **Figure 7**).

#### 4.6.1 The role of SNAD1 in innate immunity in larvae

While investigating the modulation of host aryl hydrocarbon receptor (AhR) signalling during *Pseudomonas aeruginosa* infection in zebrafish larvae, Moura-Alves et al. found that SNAD1 was upregulated 24 hours post-infection as a result of AhR signalling in the host response to the presence of bacterial quorum sensing signals (34). A similar response pattern was observed in larvae infected with isolates of *P. aeruginosa*. It was found that SNAD1 was upregulated at 3 dpi in infected larvae compared to uninfected controls (35). Moreover, in study analysing the regulation of genes associated with galanin, a biologically active neuropeptide, in zebrafish larvae after 4 days of *Mycobacterium marinum* infection, SNAD1 was found to be upregulated in response to infection in galanin+/+ larvae, while its expression showed no change in galanin knock-out larvae (36). This suggest that SNAD1 responses are dependent on correctly functioning cytokine and chemokine responses that are disrupted in galanin-/- zebrafish.

As part of its role in innate immunity, SNAD1 has been implicated in bacterial clearance. In the response of zebrafish larvae to *Shigella flexneri* infection, SNAD1 was upregulated not only during the acute response (6 hours post-infection, hpi) but also later, when bacterial clearance commenced, and was tightly regulated by G-protein coupled receptor 84 (37). Taken together, these results suggest that SNAD1 is a potential player in the innate immune response of zebrafish larvae and may play a crucial role in the immune response to bacterial pathogens in particular, in the very early stages of development.

#### 4.6.2 SNAD1 as a potential marker of progenitor-like 2 cells responses to the microbiome in zebrafish larvae

During the profiling of single cells from the intestines of zebrafish larvae at 6 days post-fertilization (dpf) raised in the presence or absence of bacteria to identify microbiota-dependent processes at the cellular level, Willms et al. observed extensive cellular heterogeneity within the conventional zebrafish intestinal epithelium (31). The authors identified 35 distinct transcriptional states in the intestine and created a map of cellular responses to the microbiota that revealed cell-specific microbial effects on growth, patterning, and immunity in the host. The distinct clusters were categorized into 18 cell types based on the expression of known markers. The clustering revealed two populations displaying features associated with intestinal progenitor cells. We identified one of the unknown marker of progenitor-like 2 cells as a SNAD1 gene (gene si:dkey-96g2.1). It should be emphasized that intestinal progenitor cells are capable of integrating signals from their niche and the gut lumen. This integration allows them to maintain the epithelial barrier, preventing microbial invasion of the host interior, by regulating their division and differentiation at an appropriate rate (54). Given the crucial role of evolutionarily-conserved innate immune defenses in maintaining stable host-microbiota relationships, the identification of SNAD1 as a potential marker for progenitor-like 2 cells may signify a new avenue in our understanding of the contributions of stem cell immunity to gut homeostasis, which to date is still poorly known.

#### 4.6.3 The role of SNAD1 in innate immunity in adult fish

We found SNAD1 as a gene modulator during immune responses in various studies related to innate immunity in adult fish, consistent with its observed role in larvae. Causey et al. investigated the response of adult rainbow trout following infection with *A. salmonicida* at 48 hpi, administered by injection, in which we found that SNAD1 was upregulated in the liver of infected fish (29). Moreover, bacteria are not the only pathogens that induce SNAD1 expression in adult fish. For example, upregulation of SNAD1 has also been identified in the immediate (16 hpi) response of zebrafish to fungal infection with *Mucor circinelloides* administered by injection in the head kidney and in other abdominal organs (38). In another study investigating fish spleen development in relation to age, a negative correlation was found between SNAD1 expression in this immune organ and the increasing age of grass carp, demonstrating its association with the development of the spleen (33). The expression pattern of SNAD1 in the head kidney, liver and spleen may provide an evidence for the specific role of SNAD1 in innate immune response of adult fish during response to different pathogens.

#### 4.6.4 Reinforcement of innate immunity by SNAD1 under cold conditions

We found strong evidence for the association of SNAD1 fluctuations with changing environmental temperatures. SNAD1 has been shown to be upregulated in response to cold exposure in fish, suggesting that it may play a role in boosting the innate immune response under cold conditions (7). This finding is crucial due to the possibility, that an expanded innate immunity, in addition to lower pathogenic pressures in a cold environment reduced the pressure to maintain robust secondary antibody diversification (5, 46).

During the acclimation of common carp to a temperature of 10°C, SNAD1 appears to be involved in remodeling the responses of fish towards responses more dependent on innate immunity, which is supported by the correlation of SNAD1 upregulation with changes in complement, acute phase and stress responses. This is consistent with the observation that in poikilothermic warm-adapted fish such as cyprinids and cichlids, temperature affects the efficacy of the adaptive arm of the immune response in particular, resulting in attenuation of T and B-cell responses (42). In zebrafish larvae with relatively low tolerance to low temperatures, acute exposure to extremely cold water can result in damage to the larvae. In general, temperatures below 15°C cause larvae to exhibit developmental abnormalities and lower survival rates. SNAD1 is also differentially regulated in the immediate response to lethal low temperature shock (10°C) in zebrafish larvae (39), suggesting a direct response to the thermal trigger. Moreover, in 2018, Somero proposed that RNA editing, including the deamination of cytidine to uracil, causes changes in protein sequences that lead to better adaptation to temperature change (55). A similar phenomenon was recently demonstrated in the octopus neural proteome, suggesting that such a mechanism is especially important in poikilotherms, in which RNA editing rates increase in cold conditions (56). Thus, we believe that RNA editing could be a possible mechanism by which SNAD1 participates in the temperature-dependent response. However, further studies are needed to indicate whether SNAD1 has a protective or adaptative function at low temperatures.

#### 4.6.5 Sex-based differences in SNAD1 protein level

It is well known, that significant differences in sex-specific immune responses occur in several vertebrate species (57). Results of many studies indicate, that females exhibit stronger innate, cellular, and humoral immune responses, than males (57), probably due to high demands of immune stability during oocyte growth (58). According to the data from two studies we discovered that the level of SNAD1 protein is twice as high in females than in males (40, 41). Li et al. using high-resolution mass spectrometry, demonstrated sex-based proteome differences in the hearts of a two-year-old cohort of adult male and female zebrafish (41). Similar findings were obtained by Niksirat et al., who conducted a comprehensive characterization of the zebrafish plasma proteome in 6-month-old and 1.5-year-old fish, enabling demonstration of sex-based differences (40). This strongly suggest, that SNAD1 could be a part of the mechanism responsible for the heightened immunity in females. However, further studies are needed to unravel the role of SNAD1 in this process.

#### 4.6.6 Involvement of SNAD1 in other stress responses

As SNAD1 expression was found to be modulated in the context of acute phase responses, we also noted transcriptome alternations during the stress response in fish. Phillips et al. demonstrated different responses of larvae exposed to 4-nonylphenol, triclosan, and triclocarban, which are endocrine disruptors that affect neurological and cardiovascular development and lipid metabolism, and the magnitude of its expression is correlated with alterations in the expression of genes involved in stress response pathways (59). Analyzing these results, we found a downregulation of SNAD1 transcripts following triclocarban exposure at a high concentration (10 nM) in zebrafish larvae. Moreover, SNAD1 downregulation has been observed in the context of the lethal progressive mitochondrial pathology related to mia40a, an evolutionarily conserved oxidoreductase which drives the biogenesis of cysteine-rich mitochondrial proteins (45). The knockout of mia40a in zebrafish larvae results in the appearance of abnormalities in pancreas development due to mutations in this gene (45), which is essential for mitochondrial intermembrane space assembly (MIA) pathway signaling and involved in the regulation of the stress response (60). These findings suggest that SNAD1 could also be considered an indicator of the stress response in fish.

#### 4.6.7 Involvement of SNAD1 in adaptive immunity

We found some indication that SNAD1 may be involved in adaptive immunity, although the contribution is not yet fully understood. Furthermore, the contribution of SNAD1 to adaptive immunity is more difficult to evaluate than its contribution to innate immunity without targeted SNAD1 knockout studies. One study that examined the plasma proteome response of rainbow trout immunized with adjuvanted hen egg-white lysozyme found alterations in the levels of SNAD1, alongside several other proteins associated with immune function (28). Interestingly, SNAD1 showed a biphasic pattern of upregulation, with upregulation shortly after immunization (7 days) and then again after 84 days when antibody responses reached their highest level (28). This result suggests that SNAD1 could be a potential marker of the response to vaccination. This hypothesis is supported by a study that analysed intestinal mucosal immunity of grass carp after DNA vaccination against the bacterium *Vibrio mimicus*, in which genes encoding SNAD1 were among the immune markers that were regulated in response to the vaccine (32). Elucidation of the exact function of SNAD1 might be slightly complicated by the fact that the genes encoding SNAD1 variants in fish do not always show the same expression pattern. For example, the expression levels of KF82305.1 and KTF82307.1, which both are accession numbers of SNAD1 sequences were found to be down- and upregulated, respectively, in response to immunization (32).

#### 4.6.8 Functional integration of SNAD1 in immunity

The involvement of SNAD1 in both innate and adaptive immunity raises questions about its specific functions in these processes. One possibility is that SNAD1 targets genomic DNA to facilitate immune receptor or antibody diversification, but this seems contradictory to the secretory nature of SNAD1 proteins. Another potential mechanism could involve the regulation of gene expression, but it remains unknown whether SNAD1 specifically targets host genes or affects pathogen genes. Pre-vertebrate animals, such as protochordates, have immune receptors belonging to the immunoglobulin superfamily. While they lack AID, they do appear to have SNADs, which are AID-like enzymes. In vertebrates, particularly those with effective high body temperature maintenance, a striking pattern of SNAD gene loss is observed. However, SNADs are present in basal members of the same vertebrate lineages that are either poikilothermic or have lower body temperatures, which may indicate their involvement in the defence system against pathogens that specifically attack organisms with lower body temperatures, perhaps to compensate for the lower activity of the adaptive arm of the immune response in these organisms (4). Alternatively, SNAD1 may target microRNAs that play a crucial role in regulating immune responses. This could be facilitated by the delivery of SNAD1 via extracellular vesicles (EVs), which would reconcile the secretory nature of these proteins with their potential involvement in diverse intracellular processes. Moreover, another possibility is that SNADs are delivered from the plasma into virally infected cells, perhaps via endocytosis, where they exert antiviral activity by mutating viral genomes, similar to the observed activity of mammalian APOBEC3 enzymes against HIV (46).

The investigation of all of these hypotheses should reveal the mechanism of SNAD1 action in fish innate and adaptive immunity and identify the position SNAD proteins within the described structure of the fish immune system.

### 4.7 Unraveling the multifaceted role of SNAD1 variants in carp’s environmental adaptation and pathogen response

The discovery of multiple SNAD1 variants suggests a complex and potentially varied role in fish biology. The exact functions and interactions of these different SNAD1 variants are still unknown. Therefore, our study serves as an initial step in this exploration, highlighting the need for further experiments to fully understand the diversity and functional implications of multiple SNAD1 variants in fish (e.g. carp). Such investigations will be crucial in unraveling the comprehensive role of SNAD1 in fish physiology and its adaptative responses to environmental changes.

Our study revealed that several SNAD1 gene variants responded differently to environmental stressors and pathogenic challenges, underscoring the SNAD1 genes' complex regulatory mechanisms and their potential role in the carp’s adaptative responses. This dual sensitivity to infections and temperature changes highlights the importance of SNAD1 in the carp’s response to environmental changes and pathogenic challenges, possibly contributing to thermotolerance and immune defense mechanisms.

This study’s findings underscore the SNAD1 gene family’s complexity and its potential contributions to the common carp’s adaptative strategies. At present, we can only speculate that the differential expression of SNAD1 variants in response to infection and temperature stressors reflects a sophisticated network of gene regulation, providing insights into the genetic mechanisms underlying stress resilience and pathogen resistance in aquatic organisms - highly characteristic of common carp, one of the most evolutionarily successful fish species, ubiquitous in multiple environments (61). Further research into the specific functions and regulatory pathways of these SNAD1 variants will be crucial in understanding their roles in the common carp’s biology and potential applications in aquaculture and conservation efforts.

## 5 Conclusions

The occurrence and importance of SNAD1 proteins in fish have not yet been fully elucidated. The identification of SNAD1 in the bodily fluids of fish, such as blood and seminal plasma, provides evidence of its secretory nature. However, the biochemical characteristics, catalytic activity, and biological functions of SNADs remain unknown and represent possible avenues for further investigations.

Our analysis provides strong evidence of the universal presence of SNAD proteins/transcripts in fish, in which expression commences after hatching and is highest in anatomical organs linked to the immune system. Moreover fish SNADs probably possess deaminase activity and are thus may deaminate nucleic acids. While the biological roles of SNADs are currently poorly understood, they are likely primarily associated with immunological processes, including innate and adaptive responses. Additionally, SNAD1 may participate in acclimation to cold conditions and sexual dimorphism. Although the precise mechanism of SNAD1 action remains unclear, it may target genomic DNA or microRNAs associated with immune response pathways, and its involvement in intracellular processes could potentially be mediated by delivery in extracellular vesicles.

Our experimental findings demonstrate dual sensitivity of SNAD1 to environmental and pathogenic pressures not only underscoring the important role in the adaptative strategy of carp but also highlights its potential as a key player in enhancing thermotolerance and immune defense mechanisms.

The lack of knowledge regarding the biological roles of SNAD means that this could be an exciting new area of research. Many questions remain unanswered, and there is an urgent need for further studies. Research is needed to determine the structures of SNADs, elucidate their mechanisms of action, identify their *in vivo* nucleic acid targets, characterize the mechanisms by which expression is regulated, and identify their relevant posttranslational modifications. Due to the possible role of SNADs in immunology, there is a need to understand their functions in innate and/or adaptive immunity and, possibly, defence against pathogens. Studies of SNADs could reveal how the mechanisms of innate and adaptive immunity intersect with the response to cold conditions. Moreover, more detailed studies of the relationship between SNAD1 and sex could reveal specific sex-dependent differences in the innate immune system.

## Data availability statement

The original contributions presented in the study are included in the article/**Supplementary Material**. Further inquiries can be directed to the corresponding author.

## Ethics statement

The animal study was approved by Animal Experiments Committee in Olsztyn, Poland (no. 93/2011), the Local Ethical Commission in Krakow, Poland with allowance (no. 49/2020), the Local Ethical Committee in Lublin, Poland (no. 32/2020) and the Lower Saxony State Office for Consumer Protection and Food Safety (LAVES), Oldenburg, Germany (no. 33.19–425 2-04-16/2144). The study was conducted in accordance with the local legislation and institutional requirements.

## Author contributions

AM: Conceptualization, Methodology, Visualization, Writing – original draft, Writing – review & editing. MD: Conceptualization, Writing – review & editing. LB: Methodology, Visualization, Writing – review & editing. MA: Methodology, Visualization, Writing – review & editing. MF: Writing – review & editing. AC: Conceptualization, Supervision, Writing – original draft, Writing – review & editing.

## Supplementary material

Supplementary Table 1 Gene names and sequences of the primers used for real-time quantitative reverse transcriptase polymerase chain reaction (qRT-PCR).
