# Supplementary material for: Secreted novel AID/APOBEC-like deaminase 1 (SNAD1) – a new important player in fish immunology
Source: Front Immunol. 2024 Mar 27;15:1340273. doi: 10.3389/fimmu.2024.1340273 (PMC11004436; doi:10.3389/fimmu.2024.1340273)
Supplement: Supplementary Table 1 — Gene names and sequences of the primers used for real-time quantitative reverse transcriptase polymerase chain reaction (qRT-PCR). [file Table_1.docx]

| **Gene Name** | **Species** | **LOC** | **Protein** | **mRNA** | **Primer Name** | **Primer sequence** |
| --- | --- | --- | --- | --- | --- | --- |
| cc_snad1_318 | Cyprinus carpio | LOC109045318 | XP_018918723 | XM_019063178 | Cc_snad1_318_qF2  Cc_snad1_318_qR2 | GGCAGAAGGACAAGGACAAGG  TTTTGGGCAGACAGAAGG |
| cc_snad1_800 | Cyprinus carpio | LOC109051800 | XP_042599546 | XM_042743612 | CC_snad1_800_qF1  CC_snad1_800_qR1 | AAGACACCAAACATTGCTCATTC  TGTAGAAAACCACACAACCATTC |
| cc_snad_409 | Cyprinus carpio | LOC109060409 | XP_042610696 | XM_042754762 | Cc_snad1_409_qF1  Cc_snad1_409_qR1 | GGCACAGGGACAATGACAGG  GCAGACCCCGACAGGAAG |
| cc_snad_506 | Cyprinus carpio | LOC109096506 | XP_018965652 | XM_019110107 | Cc_snad1_506_qF1  Cc_snad1_506_qR1 | TGAAGGGTCTCATCGTCCTC  TTAGCAAGAGCGGCAGTGTTC |
| cc_snad1_063 | Cyprinus carpio | LOC109107063 | XP_042596170 | XM_042740236 | Cc_snad1_063_qF2  Cc_snad1_063_qR2 | AAAGTGTGGTGAATGAAAATGTGG  GAAAGTTGTGTCGTGATGGATAG |
| cc_snad1_769 | Cyprinus carpio | LOC109107769 | XP_018976522 | XM_019120977 | Cc_snad1_769_qF2  Cc_snad1_769_qR2 | TCTGCCTGCTTCATCTGTGTG  CATCAGGGTGGCTGGTTGG |
| cc_snad1_962 | Cyprinus carpio | LOC122138962 | XP_042590618 | XM_042734684 | Cc_snad1_962_qF2  Cc_snad1_962_qR2 | CAGTGAGACGATCACAACTATTTAAGG  CCAGATGAAGCAGGACGATG |
| cc_snad1_946 | Cyprinus carpio | LOC122141946 | XP_042606770 | XM_042750836 | Cc_snad1_946_qF2  Cc_snad1_946_qR2 | GCTGGCCTGGAGAACTGG  CAGTCACACAGCGGTAGAGAGG |
| cc_snad1_835 | Cyprinus carpio | LOC122147835 | XP_042627763 | XM_042771829 | Cc_snad1_835_qF2  Cc_snad1_835_qR2 | TGCAGTGTCTCATCGTCCTC  TAGCAAGGGCAGCAGGGTTC |
| cc_snad1_810/cap31 | Cyprinus carpio | LOC109070810 | XP_042609326 | XM_042753392 | Cyca_ CAP31_qF3  Cyca_ CAP31_qR2 | GAACCGCCGATGTGATGAAG  CCATAGTTGGAACGCTGATG |
| cc_snad1_448 | Cyprinus carpio | LOC122140448 | XP_042600158 | XM_042744224 | Cc_snad1_448_qF1  Cc_snad1_448_qR1 | CCCTTCTCCTTGTCTCTCTGCTT  TGTTCCCTCACATTGCTTGCT |
| cc_snad1_208 | Cyprinus carpio | LOC109068208 | XP_042596168 | XM_042740234 | Cc_snad1_208_qF1  Cc_snad1_208_qR1 | GGGCCTGGAGAACTGGAAAG  GAGAGGAACATGAGCCACGA |
| cc_snad1_160 | Cyprinus carpio | LOC109104160 | XP_042627685 | XM_042771751 | Cc_snad1_160_qF1  Cc_snad1_160_qR1 | GCGACCAGGGACCACAAC  GCATAACATTGATTCTCACTCACACA |
