# Supplementary material for: Secreted novel AID/APOBEC-like deaminase 1 (SNAD1) – a new important player in fish immunology
Source: Front Immunol. 2024 Mar 27;15:1340273. doi: 10.3389/fimmu.2024.1340273 (PMC11004436; doi:10.3389/fimmu.2024.1340273)
Supplement: Supplementary file 2 [file DataSheet_1.docx]

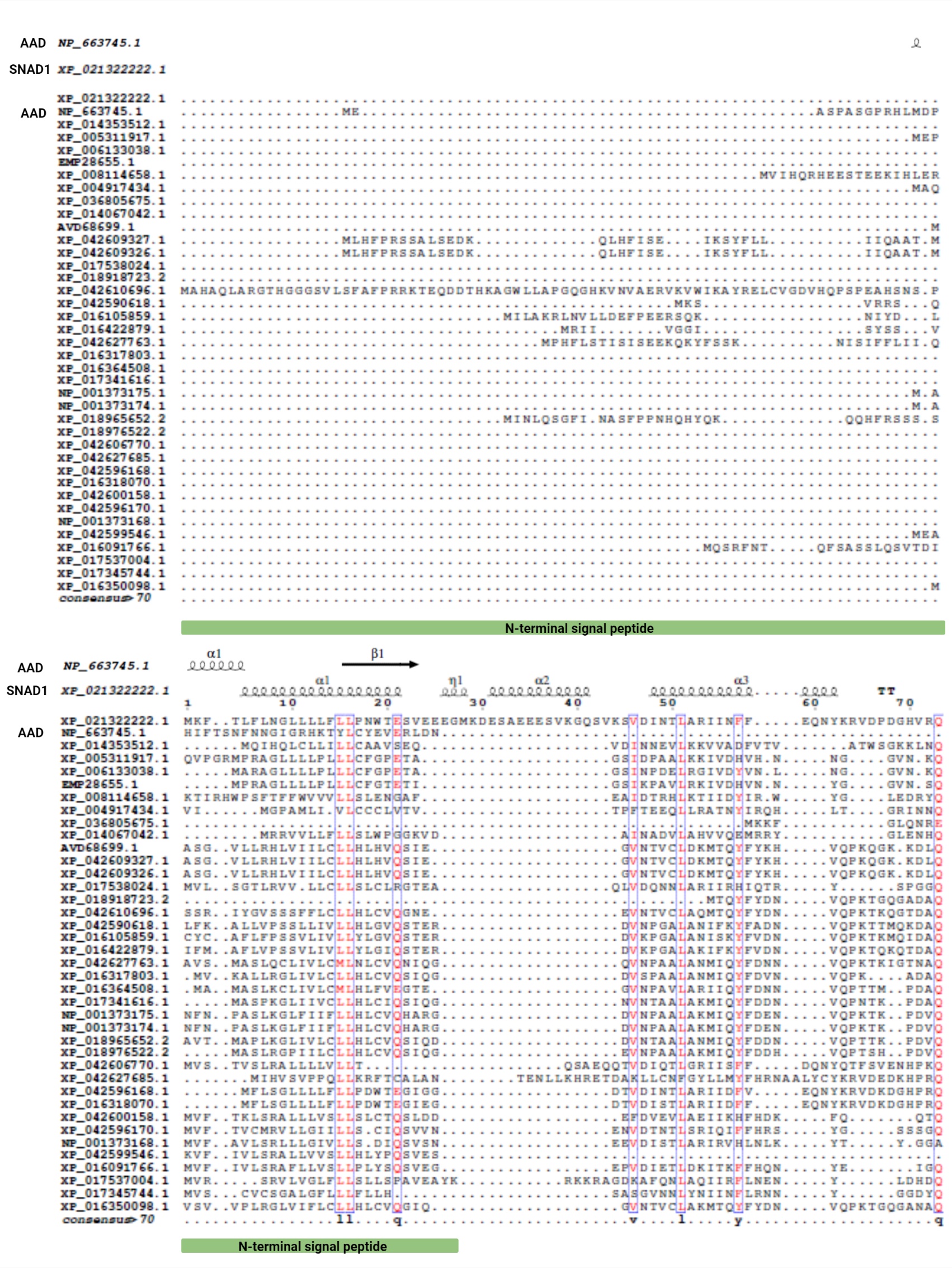


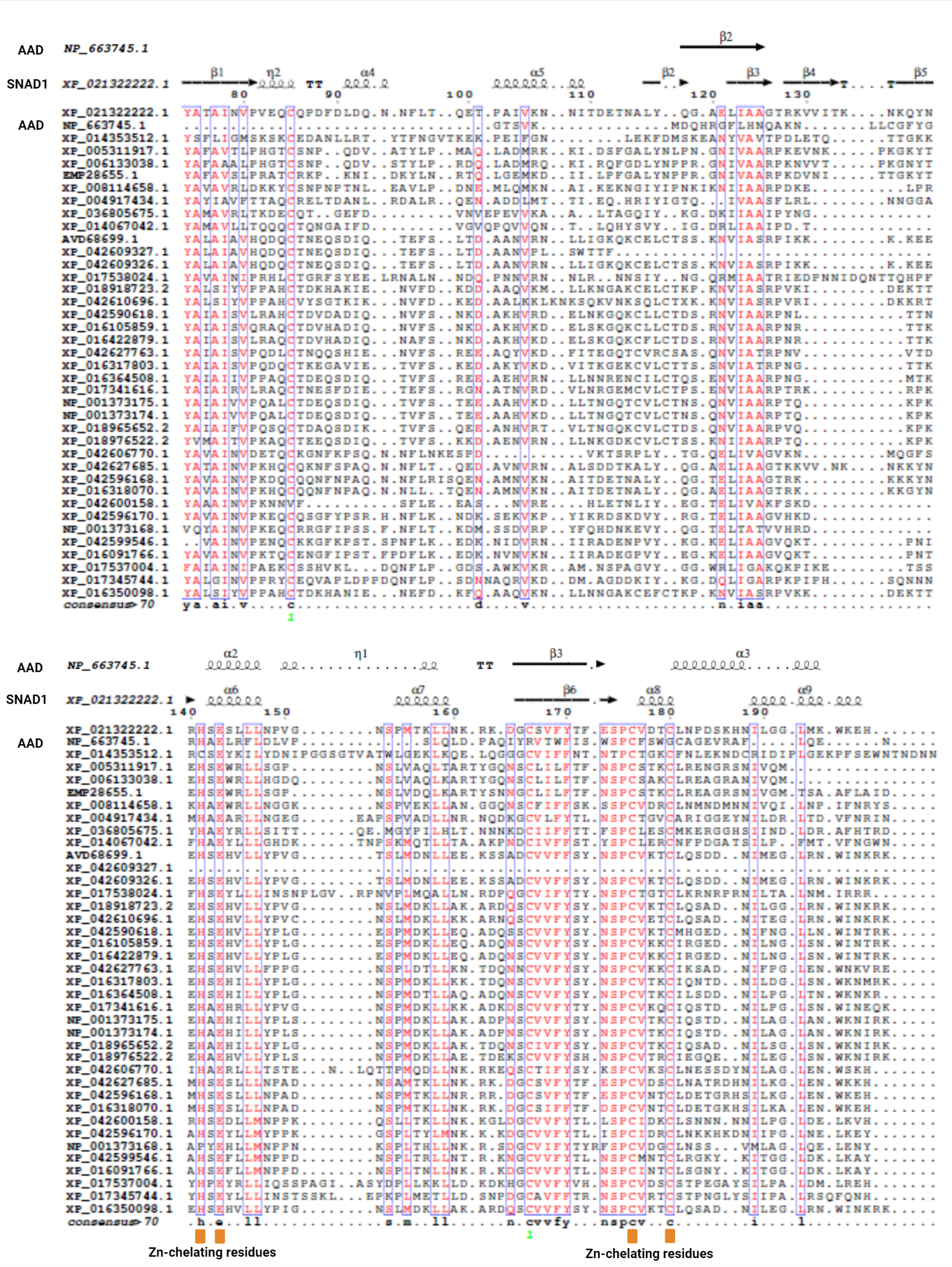


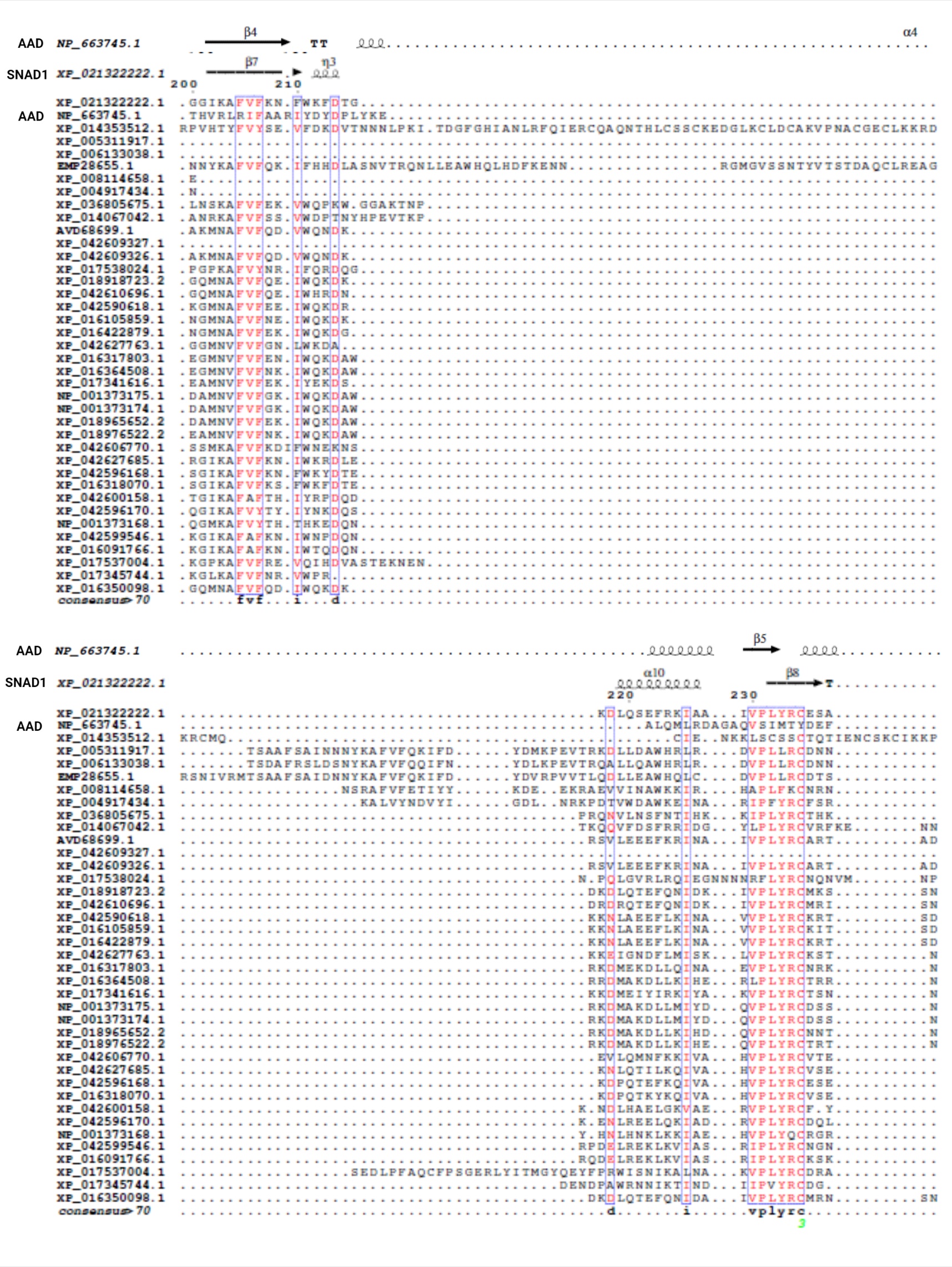


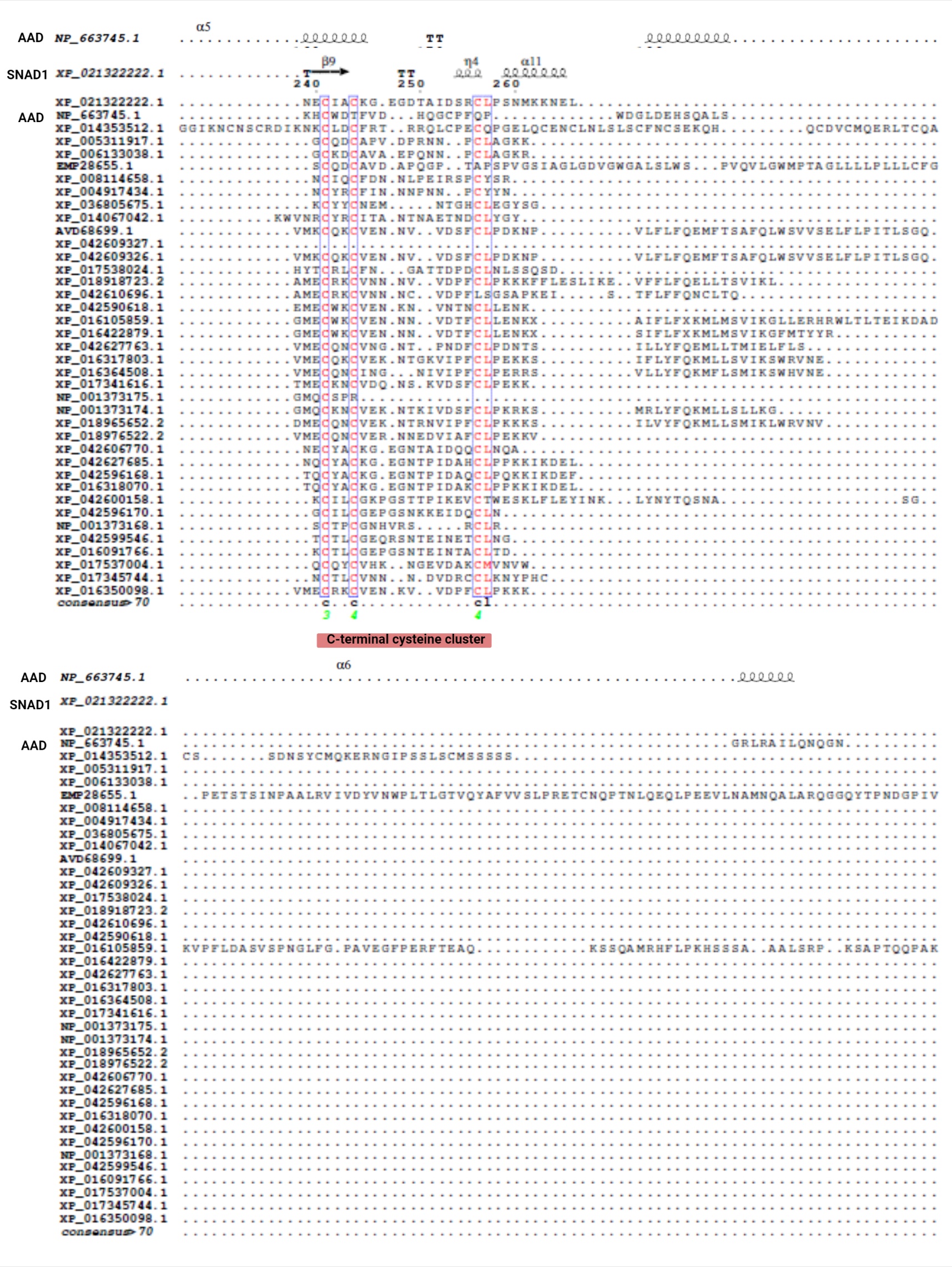


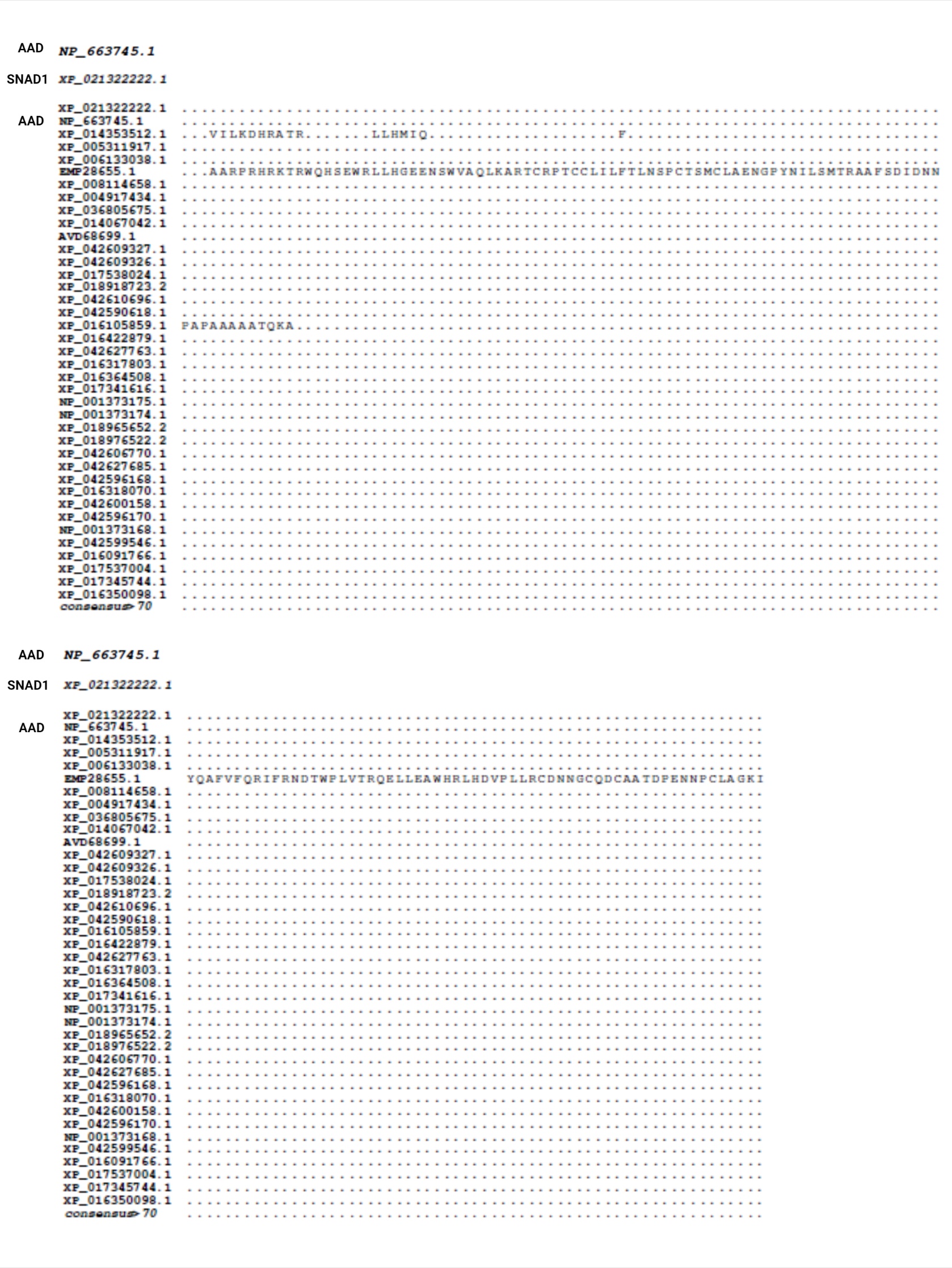


**Supplementary Figure 1.** **Primary amino acid sequence alignment of all predicted SNAD1 members identified in our phylogenetic analysis (see Figure 3, the main text) and AAD representant – human APOBEC3A.** To generate and visualize the alignment we used Clustal Omega available at <https://www.ebi.ac.uk/Tools/msa/clustalo/> and ESPript 3.0 program available at <https://espript.ibcp.fr/ESPript/cgi-bin/ESPript.cgi> . The location of conserved residues is shown in red (vertical stripes; similarity score 0.7). Secondary structures forming α helices and β sheets, generated for both SNAD1 (XP_021322222.1 from *Danio reiro*) and AAD (APOBEC3A, PDB: 5SWW) representants are indicated above the text. Structural elements, such as N-terminal signal peptide, C-terminal cysteine cluster, and Zn-chelating residues in the catalytic center, are marked with colored rectangles below the text. (Figure created with [http://*biorender*.com](http://biorender.com))
