# Supplementary material for: Secreted novel AID/APOBEC-like deaminase 1 (SNAD1) – a new important player in fish immunology
Source: Front Immunol. 2024 Mar 27;15:1340273. doi: 10.3389/fimmu.2024.1340273 (PMC11004436; doi:10.3389/fimmu.2024.1340273)
Supplement: Supplementary file 3 [file DataSheet_2.docx]

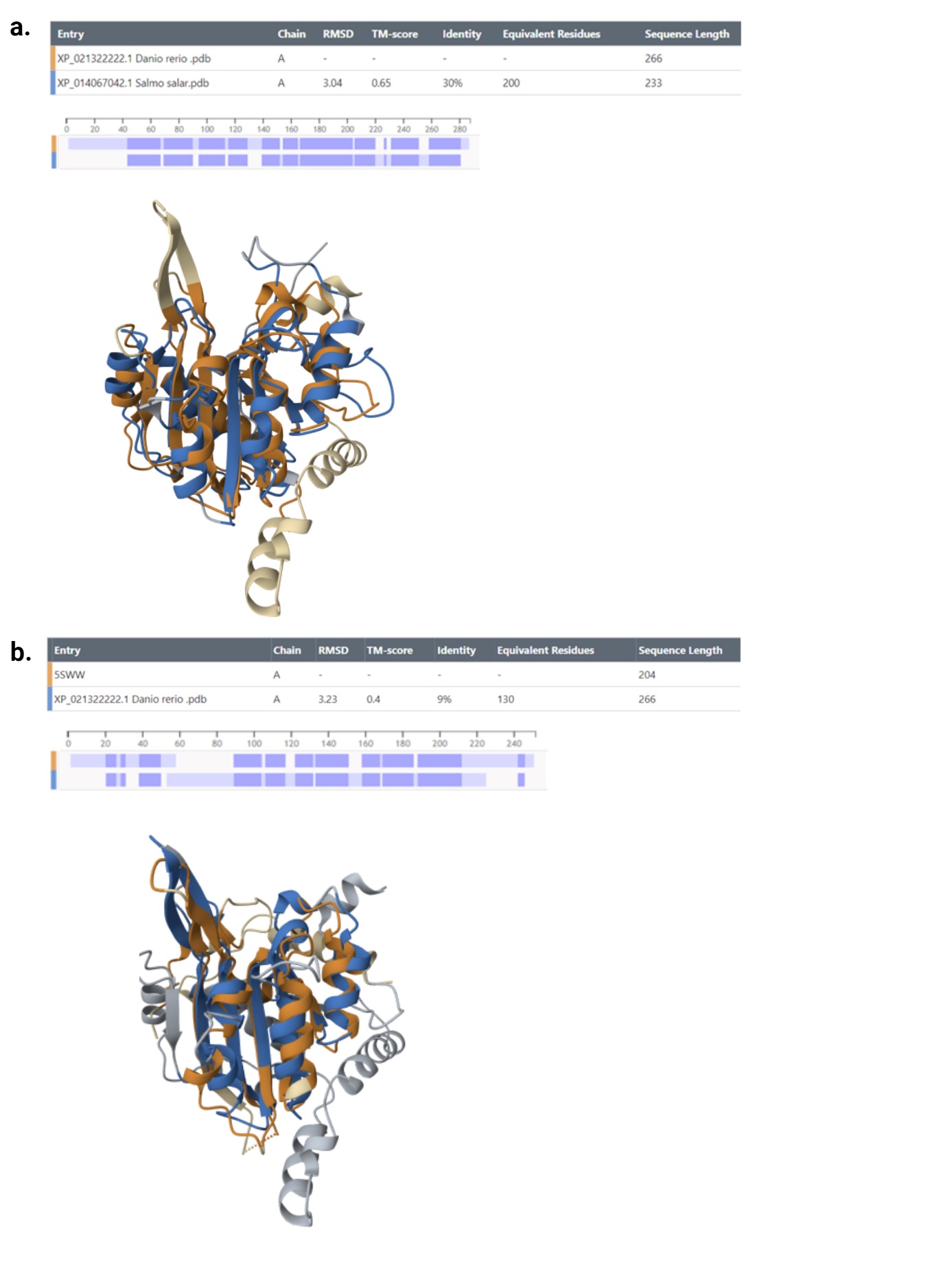


**Supplementary Figure 2.** **3D structure alignment of predicted SNAD1 members from warm-water-adapted and cold-water-adapted fish, and a comparison of the above with X-ray structure of AAD representant – human APOBEC3A.** **(a)** Superposition of modeled 3D structures of SNAD1 members from warm-water-adapted (*Danio rerio*) and cold-water-adapted fish (*Salmo salar*). For the comparison, we selected sequences (*XP_021322222.1* and XP_014067042.1 from *Danio rerio* and *Salmo salar,* respectively) that show the highest similarity. To generate the superposition, we used Pairwise Structure Alignment tool available at [www.rcsb.org/docs/tools/pairwise-structure-alignment](http://www.rcsb.org/docs/tools/pairwise-structure-alignment). Tables present basic parameters for superpositions. TM-score (template modeling score) is a measure of topological similarity and ranges between 0 and 1, where 1 indicates a perfect match and 0 is no match between structures. Is accepted that scores < 0.2 indicate unrelated proteins while > 0.5 indicate the same protein fold. When superimposed protein structures are presented in orange and blue. Regions that have no counterpart in the second structure are marked in beige or gray. **(b)** Superposition of the predicted 3D structure of SNAD1 from *Danio rerio* (XP_021322222.1) and X-ray structure of AAD member – human APOBEC3A protein (PDB 5SWW). (Figure created with http://biorender.com)
