# Supplementary material for: Secreted novel AID/APOBEC-like deaminase 1 (SNAD1) – a new important player in fish immunology
Source: Front Immunol. 2024 Mar 27;15:1340273. doi: 10.3389/fimmu.2024.1340273 (PMC11004436; doi:10.3389/fimmu.2024.1340273)
Supplement: Supplementary file 4 [file DataSheet_3.docx]

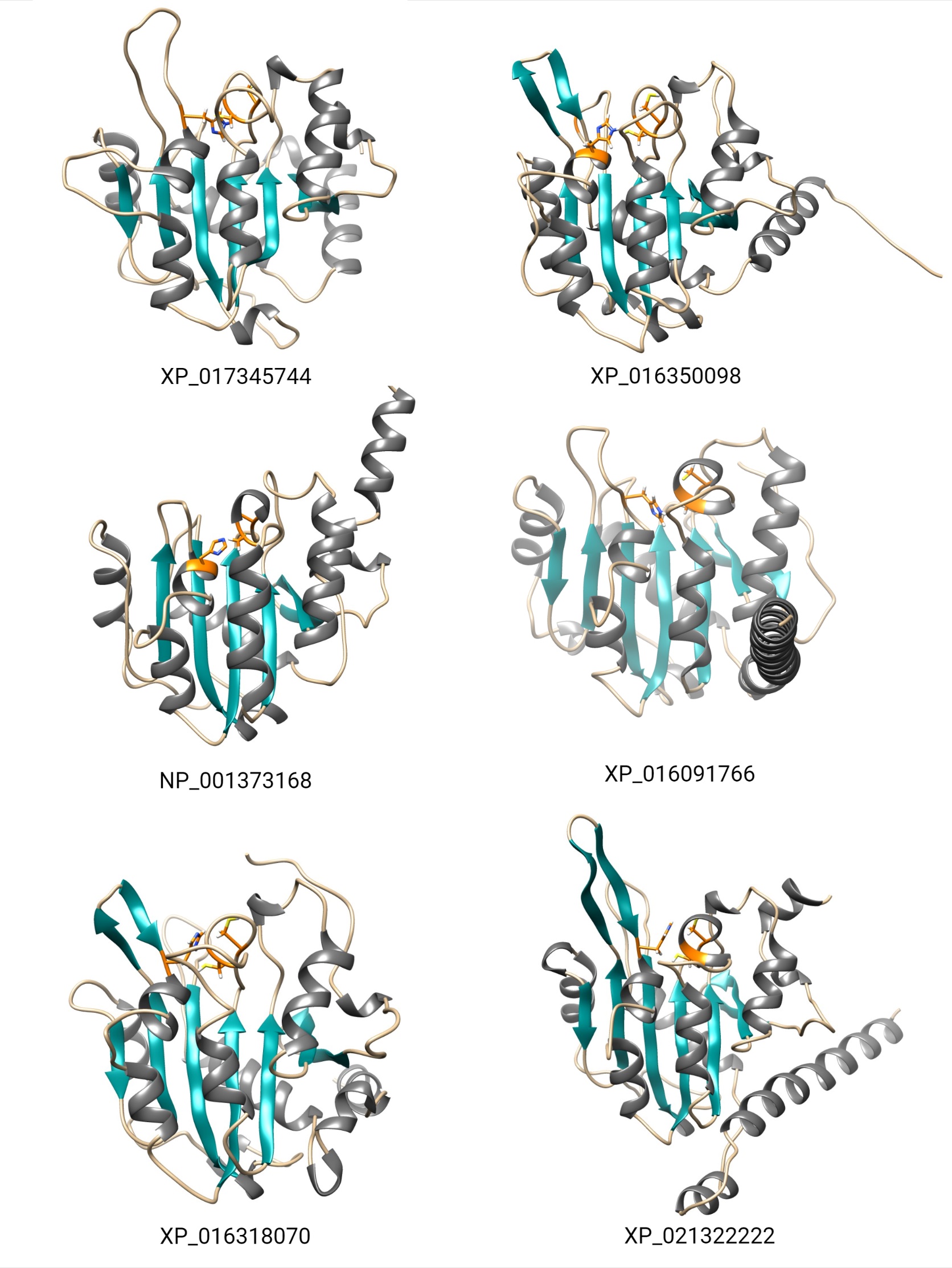


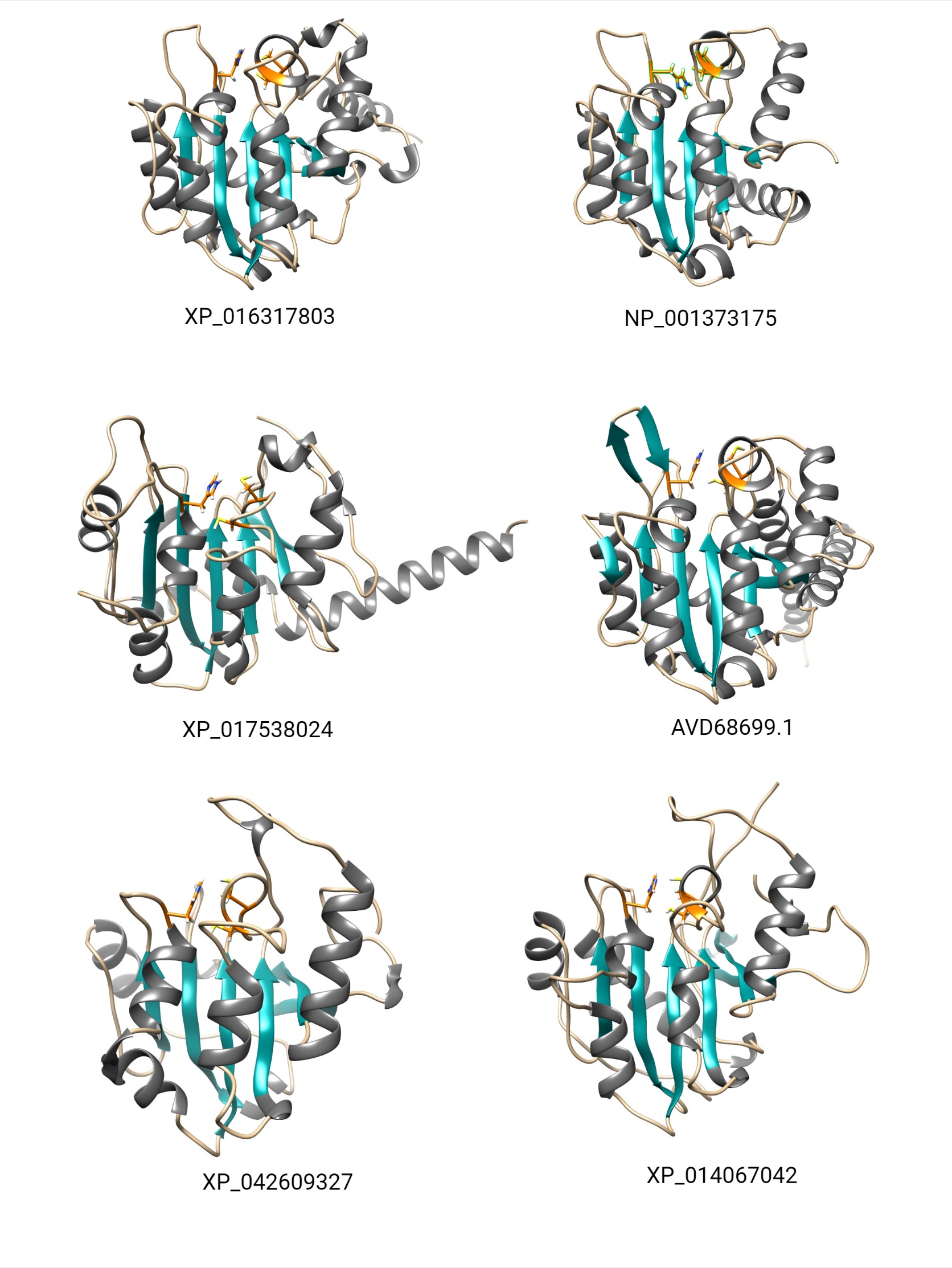


**Supplementary Figure 3. 3D structural models (generated using the RoseTTAFold method) of selected SNAD1 members from various fish species.** For 3D structure predictions, we selected 12 sequences – 2 sequences for each of the 6 identified phylogenetic groups marked in Figure 3 (main text) in different colors. The zinc-coordinating motif in the predicted catalytic centers is shown in orange; β strands are shown in cyan; α helices are shown in grey (Figure created with [http://*biorender*.*com*](http://biorender.com))
